# Metabolomic and transcriptomic signatures of influenza vaccine response in healthy young and older adults

**DOI:** 10.1111/acel.13682

**Published:** 2022-08-23

**Authors:** Chih‐Hung Chou, Subhasis Mohanty, Heather A. Kang, Lingjia Kong, Julian Avila‐Pacheco, Samit R. Joshi, Ikuyo Ueda, Lesley Devine, Khadir Raddassi, Kerry Pierce, Sarah Jeanfavre, Kevin Bullock, Hailong Meng, Clary Clish, Fabio R. Santori, Albert C. Shaw, Ramnik J. Xavier

**Affiliations:** ^1^ Broad Institute of MIT and Harvard Cambridge Massachusetts USA; ^2^ Section of Infectious Diseases, Department of Internal Medicine Yale School of Medicine New Haven Connecticut USA; ^3^ Department of Laboratory Medicine Yale School of Medicine New Haven Connecticut USA; ^4^ Department of Neurology Yale School of Medicine New Haven Connecticut USA; ^5^ Department of Pathology Yale School of Medicine New Haven Connecticut USA; ^6^ Center for Molecular Medicine University of Georgia Athens Georgia USA; ^7^ Klarman Cell Observatory Broad Institute of Harvard and MIT Cambridge Massachusetts USA; ^8^ Center for Computational and Integrative Biology and Department of Molecular Biology Massachusetts General Hospital and Harvard Medical School Boston Massachusetts USA

**Keywords:** immune response, influenza, metabolomics, systems biology, systems vaccinology, transcriptomics, vaccine

## Abstract

Seasonal influenza causes mild to severe respiratory infections and significant morbidity, especially in older adults. Transcriptomic analysis in populations across multiple flu seasons has provided insights into the molecular determinants of vaccine response. Still, the metabolic changes that underlie the immune response to influenza vaccination remain poorly characterized. We performed untargeted metabolomics to analyze plasma metabolites in a cohort of younger and older subjects before and after influenza vaccination to identify vaccine‐induced molecular signatures. Metabolomic and transcriptomic data were combined to define networks of gene and metabolic signatures indicative of high and low antibody response in these individuals. We observed age‐related differences in metabolic baselines and signatures of antibody response to influenza vaccination and the abundance of α‐linolenic and linoleic acids, sterol esters, fatty‐acylcarnitines, and triacylglycerol metabolism. We identified a metabolomic signature associated with age‐dependent vaccine response, finding increased tryptophan and decreased polyunsaturated fatty acids (PUFAs) in young high responders (HRs), while fatty acid synthesis and cholesteryl esters accumulated in older HRs. Integrated metabolomic and transcriptomic analysis shows that depletion of PUFAs, which are building blocks for prostaglandins and other lipid immunomodulators, in young HR subjects at Day 28 is related to a robust immune response to influenza vaccination. Increased glycerophospholipid levels were associated with an inflammatory response in older HRs to flu vaccination. This multi‐omics approach uncovered age‐related molecular markers associated with influenza vaccine response and provides insight into vaccine‐induced metabolic responses that may help guide development of more effective influenza vaccines.

## Graphical abstract

Seasonal influenza causes respiratory infections and significant morbidity, especially in older adults, and response to flu vaccine remains particularly poor among this population. We performed untargeted plasma metabolomics and transcriptomics in a cohort of younger and older subjects before and after influenza vaccination. We identified age‐ and vaccine‐related molecular markers associated with vaccine response, insights which may guide development of improved influenza vaccines.

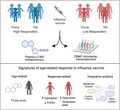

**Abbreviations**
BTM: blood transcription module
DAMs: differentiatlly abundant metabolites
HA: hemagglutinin antigen
HAI: hemagglutination inhibition
HR: high responder
IFN: interferon
LR: low responder
maxRBA: maximum residual after baseline adjustment
MUFA: monounsaturated fatty acids
PBMC: peripheral blood mononuclear cells
PC: phosphatidylcholine
PE: phosphatidylethanolamine
PUFA: polyunsaturated fatty acids
SFA: saturated fatty acids
sPLS: sparse partial least squared
TAG: triacylglycerol

## 1 BACKGROUND

Seasonal epidemics caused by influenza viruses, such as H1N1, H3N2 influenza A viruses, and influenza B viruses, are a major public health concern that cause mild to severe respiratory infections in humans. A recent study estimated that up to 5 million cases and 290,000 to 650,000 influenza‐associated respiratory deaths annually occur worldwide, particularly among adults aged 65 or older (Iuliano & Roguski, 2018). Avian influenza viruses (H5N1, H7N9, and others) may cross the species barrier through a re‐assortment step to cause pandemics in the human population that have increased morbidity and mortality relative to seasonal influenza epidemics (Krammer, 2019; Palese, 2004; Smith et al., 2009). To decrease the public burden caused by seasonal or pandemic influenza, three types of effective vaccines (inactivated, live attenuated, and recombinant hemagglutinin antigen [HA] vaccines) have been developed. However, response to the vaccine remains poor in many populations. Older populations, for example, have a diminished capacity to mount a sufficient protective response to flu vaccination due to age‐related changes in immune system function (Grubeck‐Loebenstein & Della Bella, 2009; McElhaney, 2011; Poland et al., 2014).

Improvement of vaccine efficacy requires an understanding of the mechanisms by which vaccination bolsters the immune response. In response to challenges such as infection and vaccination, immune cells undergo metabolic reprogramming to address increased energy demands and need for rapid effector cell proliferation (Jung et al., 2019). In recent years, studies from the emerging field of immunometabolism have indicated that cellular metabolism plays a critical role in immune cell differentiation, development, and maintenance (Haschemi & Kosma, 2012; O'Neill et al., 2016). Several key metabolic pathways have been linked to control of immunity, including glycolysis (Shi et al., 2011), TCA cycle (Liu et al., 2017; Tannahill et al., 2013), pentose phosphate pathway (Haschemi & Kosma, 2012), fatty acid metabolism (Berod & Friedrich, 2014), and metabolism of amino acids such as glutamine (Nakaya et al., 2014) and tryptophan (Uyttenhove et al., 2003). The development of highly sensitive metabolic profiling techniques has facilitated the identification of metabolite signatures associated with immunological responses, providing valuable tools for dissecting complex immunometabolic signaling networks (Haschemi & Kosma, 2012; O'Neill et al., 2016).

Studies of systems vaccinology in influenza have used cellular and transcriptomic profiles of peripheral blood mononuclear cells (PBMC) and serology to predict the immune response to influenza vaccination in healthy adults (Bucasas & Franco, 2011; Furman & Jojic, 2013; Nakaya et al., 2011; Obermoser et al., 2013; Tsang et al., 2014). A common finding among these studies is that immune response to influenza vaccine can be predicted by early response (Days 1–3) post‐vaccination and is associated with a transcriptomic signature enriched for gene ontologies for innate immune responses, antigen presentation, and induction of interferon type I response genes. Longitudinal studies showed consistent transcriptomic and microRNA signatures of influenza vaccination across seasons in the same cohort (Nakaya et al., 2015). One of the most consistent findings is that age is an important contributor to influenza vaccine response (Furman & Jojic, 2013; Haschemi & Kosma, 2012; Nakaya et al., 2011; Rogers et al., 2019; Tsang et al., 2014). We previously investigated the transcriptomic signature of immune response to influenza vaccination and found that a mitochondrial biogenesis signature was associated with vaccine antibody response (Thakar et al., 2015). A 5‐year longitudinal study led to the identification of age‐related transcriptional signatures that are predictive of flu vaccination responses (Avey & Mohanty, 2020; HIPC‐CHI Signatures Project Team and HIPC‐I Consortium, 2017). The first multi‐omics integrated analysis of metabolomics, transcriptomics, and gut microbiome composition uncovered associations between bacterial species and metabolic phenotypes in healthy and antibiotic‐treated adults receiving influenza vaccine, demonstrating that antibiotic‐induced microbiome changes affect the human immune response to influenza vaccine (Hagan & Cortese, 2019). These studies demonstrate the importance of identifying molecular markers associated with vaccine response to help identify potential avenues for improvement of vaccine efficacy.

In this study, we use a combination of transcriptomics and untargeted metabolomics to define signatures of high and low antibody response after vaccination against influenza in a cohort of young and older adults. Consistent with previous studies, we also identify age as one of the main factors that determines response to influenza vaccination. The difference in response between age groups was associated with signatures derived from metabolic pathways involved in fatty acid and sterol lipid metabolism. Young high responders showed increased tryptophan pathway metabolites and reduced levels of polyunsaturated fatty acids. In contrast, older high responders show increased fatty acid synthesis and accumulation of cholesteryl esters. Through this multi‐omics approach, we identify age‐related molecular markers associated with influenza vaccine response. Uncovering these molecular profiles will help to identify populations at risk of influenza vaccine failure, as well as to inform design of vaccine development trials to improve vaccine efficacy.

## 2 RESULTS

### 2.1 Age has the strongest effect on the metabolic response to influenza vaccination

**FIGURE 1:**
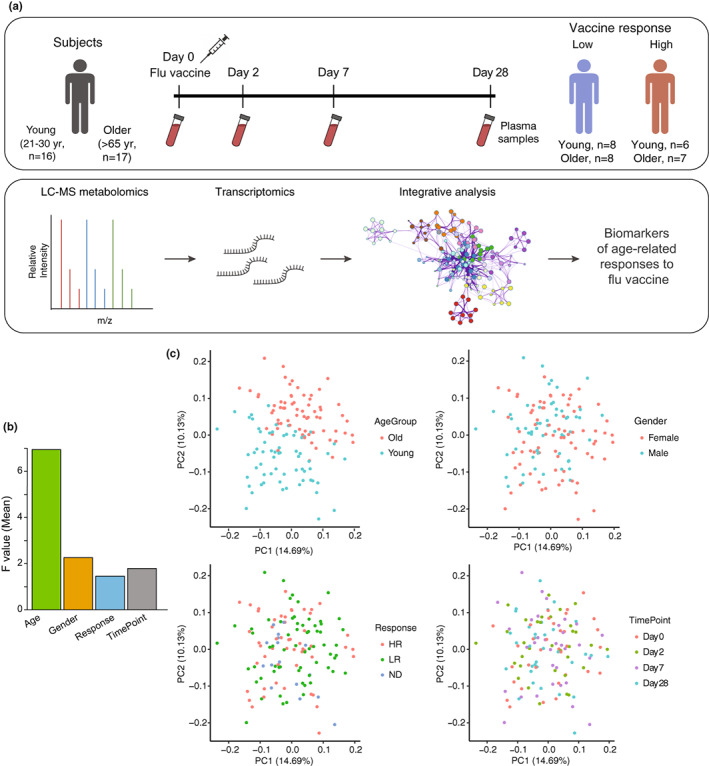
Study design and global characterization of plasma metabolome profiles. (a) Blood plasma samples from a total of 33 subjects (16 young (age 21–30 years) and 17 older (age ≥65)) were collected prior to vaccination (Day 0) and at 2, 7, and 28 days post‐vaccination. Subjects were classified as high responders (HR) or low responders (LR) by HAI titers (see Section 4) at 28 days post‐vaccination using maxRBA (Avey & Mohanty, 2020). Metabolite abundance in plasma was assessed using high‐throughput profiling by LC–MS‐based metabolomics, and gene expression levels in PBMCs were assessed using high‐throughput profiling by Illumina beadChips microarray as previously reported (Thakar et al., 2015). Finally, computational and integrative analyses of the metabolomics and transcriptomics datasets were performed. (b) The effect of factors such as age, gender, response, and timepoint and their interactions on the variation of plasma metabolite abundance in the study cohort. *F* value determined by one‐way ANOVA. (c) Sampleto‐sample variation in plasma metabolomics revealed by principal component analysis (PCA). Each dot represents a sample, colored by age, gender, response, and time point, respectively.

To assess metabolic changes after flu vaccination, we performed untargeted metabolomics on blood plasma samples of 33 individuals of a well‐studied cohort of young (age 21–30) and community‐dwelling older (age ≥65) adults (Thakar et al., 2015; Figure 1a) who received the seasonal trivalent inactivated influenza vaccine (TIV, A/California/7/09 (H1N1)‐like virus; A/Perth/16/2009 (H3N2); and B/Brisbane/60/2008) during the 2011–2012 season. These 33 subjects were selected for this study based on their classification as either strong or weak responders to influenza vaccine and availability of transcriptomic data. The subjects' blood plasma samples were collected pre‐vaccination (Day 0), and at Days 2, 7, and 28 post‐vaccination. Subjects were classified by response rate based on flu vaccine‐specific hemagglutination inhibition (HAI) titers, which were measured at Day 0 and at 28 days post‐vaccination. To account for the effect of pre‐vaccine HAI titer, we calculated the maximum residual after baseline adjustment (maxRBA) (Avey & Mohanty, 2020). The maxRBA calculation corrects for the dependence on baseline titers by modeling post‐vaccine fold increase in HAI titer as an exponential function of pre‐vaccine titer and taking the maximum residual across vaccine strains (Figure S1). Using this method, we defined high (HR) and low responders (LR) as the top and bottom 40th percentiles of the residuals, respectively. The 40th percentile was chosen because at this cutoff there is <10% indeterminate response to flu vaccine (Avey & Mohanty, 2020). This allows for an increase in sample size compared with lower percentiles without increasing substantially the number of false positives in the analysis. For this study, a subset of plasma samples from the previously described cohort was selected to adjust for differing proportions of HR/LR among young and older groups. In total, we selected samples from 16 young and 17 older subjects, with a total of 13 HR and 16 LR (6 HR and 8 LR in young subjects and 7 HR and 8 LR in older subjects) and 4 with an indeterminate response.

The untargeted metabolomic profile of plasma samples suggests that age was the largest contributor to variation in metabolite profiles, whereas gender, response to vaccine, or time point had less pronounced effects (Figure 1b). Principal component analysis (PCA) showed a clear separation of the young and older groups, consistent with the variation analysis (Figure 1c). Because of statistically significant BMI differences in the young vs. older populations (*p* = 0.006, Table 1), we included BMI as a variable in our linear regression models to account for its effect on differential metabolite abundance across age groups. At baseline (Day 0), distinct subclasses of metabolites were differentially abundant between younger and older individuals. These include fatty acids, linoleic acids, and glycerophospholipids, which were higher in the young group, while amino acids and triacylglycerols were higher in the older group (Figure S2). Based on the strong age‐dependent influence on metabolite profiles, we chose to perform subsequent analyses on young and older groups independently.

**TABLE 1:** Clinical characteristics of older and young subjects

Group	Older (*N* = 17)	Young (*N* = 16)	*p*‐Value
Age			
Median [MAD]	72 [5.9]	27 [3]	1.4e‐07
Gender			
Female	59% (10)	56% (9)	ns
Male	41% (7)	44% (7)	
Response			
HR	41% (7)	38% (6)	ns
LR	47% (8)	50% (8)	
ND	12% (2)	12% (2)	
BMI			
Median [MAD]	27.4 [3.3]	22 [1.5]	0.006
Mean [SD]	28.5 [5.7]	23.5 [3.3]	
Race			
White	82% (14)	75% (12)	ns
Asian	5.9% (1)	12% (2)	
Black or African American	12% (2)	0% (0)	
Other/Unknown	0% (0)	12% (2)	

*Note*: Continuous measures (Age and BMI): Kolmogorov–Smirnov test. Categorical measures (Gender, Response, and Race): Chi‐square test.

### 2.2 Differential molecular signatures after flu vaccination in young and elderly

**FIGURE 2:**
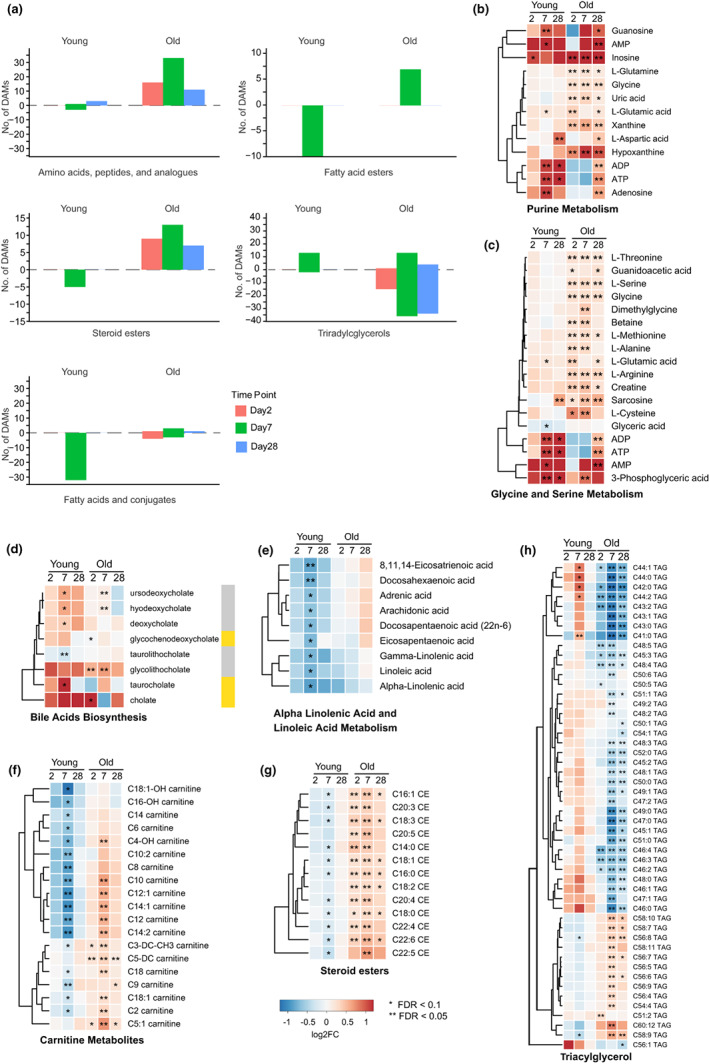
Differential molecular signatures induced by flu vaccination in young and old groups. (a) Differentially abundant metabolite levels (absolute value of fold change ≥1.2, *p* < 0.05, and FDR < 0.2) at Day 2, Day 7, and Day 28 post‐vaccination relative to Day 0 in young and old groups. Selected metabolite classes are shown. Molecular signatures in the following pathways show similar trends in young and older groups: (b) purine metabolism, (c) glycine and serine metabolism, (d) bile acid biosynthesis. Molecular signatures in the following pathways show differing trends in young and older groups: (e) alpha‐linolenic acid and linoleic acid metabolism, (f) steroid esters, (g) carnitine metabolites and (h) triacylglycerol. Results for AMP, ADP, and ATP appear in both (b) and (c) as they are members of both pathways. Color labels correspond to indicated log2FC. Gold color labels in the right of panel d represent the primary bile acids and gray color labels represent the secondary bile acids. *FDR < 0.1, **FDR < 0.05.

**FIGURE 3:**
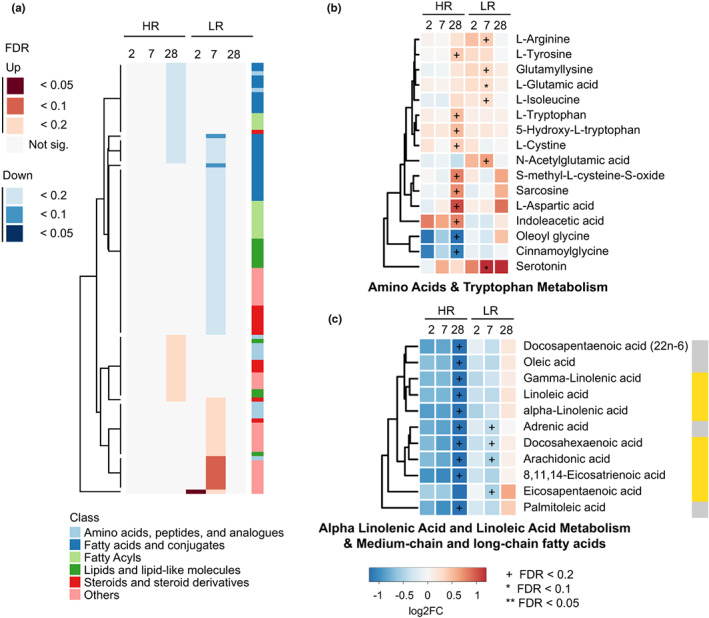
Molecular signatures associated with response to vaccination in young subjects. (a) Differentially abundant metabolite levels (absolute value of fold change ≥1.2, *p* < 0.05, and FDR < 0.2) at Day 2, Day 7, and Day 28 post‐vaccination relative to Day 0 for HR and LR groups. (b, c) Molecular signatures for the top differential metabolic pathways in young HR and LR (b) Amino acids & tryptophan Metabolism. (c) Alpha linolenic acid and linoleic acid metabolism & medium‐chain and long‐chain fatty acids. The metabolites that belong to PUFA metabolites are gamma‐linolenic acid, linoleic acid, alpha‐linolenic acid, docosahexaenoic acid, arachidonic acid, 8,11,14‐Eicosatrienoic acid, and Eicosapentaenoic acid. Gold color labels in the right of panel c represent the PUFA metabolites. Color labels correspond to indicated log2FC. ^+^FDR < 0.2, *FDR < 0.1, **FDR < 0.05.

We next compared pre‐vaccination metabolite abundances with those at Days 2, 7, and 28 after vaccination to determine differentially abundant metabolites (DAMs). We annotated 534 metabolites using the Human Metabolome Database (HMDB), which classifies metabolites by their chemical structure (Wishart et al., 2007; Figure S3a,b). Among the five groups of metabolites that contained the highest number of DAMs were amino acids, peptides, and analogues, fatty acids and conjugates, fatty acid esters, steroid esters, and triradylglycerols (a sub‐class of lipids that includes triacylglycerols) (Figure 2a). Purine metabolism (Figure 2b) and glycine and serine metabolism (Figure 2c) are the major metabolic pathways related to amino acids, nucleotides, peptides, and analogues. We observed similar post‐vaccination trends across age groups for several metabolite subclasses. For example, purine metabolism pathway metabolites significantly increased after vaccination; this increase was particularly strong among older subjects. Notably, gene expression analysis using a previously published dataset from this same cohort (Thakar et al., 2015) revealed upregulation of genes involved in the purine‐containing compound metabolic process (GO:0072521). Most of the genes upregulated at Day 7 and Day 28 were in the young group, whereas most upregulated genes at Day 2 were in the older group (Figure S4a). Inosine increased in both age groups at Day 2, and this increase persisted at Day 7 and Day 28 in the older cohort. Inosine is an intermediate in the degradation of purines and purine nucleosides that helps innate immunity distinguish cellular from viral RNA (Mannion et al., 2014) and plays a role in CD8^+^ effector T‐cell function under glucose‐restricted conditions (Wang et al., 2020). Metabolites from the glycine and serine metabolic pathway also increased after vaccination, although the majority of metabolites that changed in the young were not significant (Figure 2c). Sarcosine was significantly increased in older subjects at Days 2, 7, and 28 but only increased in young subjects by Day 28 (Figure 2c, Table S1). Production of cytosolic sarcosine can be catalyzed by glycine N‐methyltransferase (GNMT), which mediates sarcosine synthesis using S‐adenosylmethionine (SAM) (Luka et al., 2009). This conversion has been associated with decreases in energetic metabolism (Obata et al., 2014), enhancement of dendritic cell migration, and response to dendritic‐based cancer cell vaccines (Dastmalchi & Karachi, 2019). However, the possible effect of elevated levels of sarcosine in older subjects is not clear; sarcosine is elevated in older subjects but it is not a strong indicator of high versus low response to flu vaccine in this cohort (Figure 3b). 3‐phosphoglyceric acid (3‐PG), a glycolysis intermediate that is also an intermediate in serine synthesis (Figure S4b), was significantly increased in young adults after vaccination at Day 7 and Day 28 and in older adults at Day 7, and serine was significantly increased in older individuals (Figure 2c, Table S1). This correlated well with the patterns on T cell‐related blood transcription module (BTM) profiles, which were systematically constructed and integrated from publicly available human blood transcriptomes by (Li et al., 2014), in young and older adults (Figure S4c), as previous studies reported that serine was required for T‐cell proliferation to regulate adaptive immunity (Ma et al., 2017).

Among metabolites in the bile acid synthesis pathway, our data show that the secondary bile acids hyodeoxycholate and ursodeoxycholate increase after vaccination in young and older adults at Day 7. Deoxycholate increases in young subjects after vaccination at Day 7, while glycolithocholate increases in older adults at Days 2 and 7 (Figure 2d, Table S1). A previous study suggested that the loss of secondary bile acids is associated with NLRP3 inflammasome and AP‐1‐associated gene *JUN* and *FOS* activation, which may imply a negative correlation between secondary bile acids and NLRP3/JUN/FOS (Hagan & Cortese, 2019). Consistent with this, we observed downregulated gene expression for *NLRP3* post‐vaccine in young and old subjects. However, *JUN* and *FOS* expression were upregulated in older subjects (Figure S4d), an observation consistent with studies suggesting that secondary bile acids (Fu & Coulter, 2019) and AP‐1 transcription factors (Qiao et al., 2016) are associated with pro‐inflammatory signaling.

Other metabolites exhibited differences in abundance between the two age groups after vaccination. For example, metabolism of α‐linolenic and linoleic acids, as well as fatty acyl carnitines and cholesteryl esters, is significantly decreased in younger subjects on Day 7 (Figure 2e–g). In contrast, in older adults these metabolites are either unaffected (α‐linolenic and linoleic acid metabolism) or largely increased (fatty acyl carnitines and cholesteryl esters) (Figure 2e–g). Other metabolites such as triacylglycerol (TAGs) metabolites that changed significantly after vaccination in the older cohort, show the opposite trend in young adults (Figure 2h). This may reflect a differential balance in fatty acid synthesis/fatty acid oxidation in older versus younger adults.

To identify enriched gene functions from our differentially expressed genes, we analyzed BTMs. Among the most notable age‐dependent changes were the modules enriched in T cells (I) (M7.0), enriched in T cells (II) (M223), and signaling in T cells (I) (M35.0), as well as enriched in NK cells (I) (M7.2) and (II) (M157) and NK cell surface signature (S1), all of which were upregulated in the older group after vaccination (Figure S4c).

### 2.3 Molecular signatures associated with response to vaccination in young subjects

To examine changes in metabolite profiles in the context of vaccine response among young subjects, we identified DAMs in young HRs and LRs at Days 2, 7, and 28 after flu vaccination compared with baseline values. We identified a total of 103 DAMs across all time points (Figure 3a). The highest number of differentially abundant metabolites in HR belong to amino acid and tryptophan metabolism, alpha‐linolenic and linoleic acid metabolism, and metabolism of medium‐chain and long‐chain fatty acids. The differences we observed among young HR/LRs were mostly nominally significant with an FDR > 0.1, with the exception of L‐glutamic acid and serotonin. In young HRs L‐tryptophan, 5‐Hydroxy‐L‐tryptophan, and indoleacetic acid were increased after vaccination at Day 28 (Figure 3b, Table S2); this upward trend was only observed in younger and not older subjects. Since tryptophan catabolism through the kynurenine pathway suppresses T‐cell responses, the higher levels of these tryptophan metabolites could be consistent with a stronger T‐cell response in HR (Platten et al., 2019). We observed opposing trends at Day 28 in α‐linolenic acid and linoleic acid metabolism and medium‐chain and long‐chain fatty acids, with a decrease after vaccination occurring at Day 28 in young HRs (Figure 3c). This group of metabolites includes seven polyunsaturated fatty acids (PUFAs: arachidonic acid, linoleic acid, gamma‐linolenic acid, eicosapentaenoic acid, alpha‐linolenic acid, 8,11,14‐eicosatrienoic acid, and docosahexaenoic acid), most of which decrease after vaccination at Day 28 in the young HR group (except eicosapentaenoic acid) (Figure 3c, Table S2). BTM profiles of young HR and LR showed an upregulation of genes involved in dendritic cell activation (II) (M165), type I interferon response (M127), and viral sensing and immunity; IRF2 targets network (I) (M111.0 and M111.1) in HR patients at Day 2 post‐vaccination (Figure S5a). A previous study indicated that dendritic cells (DCs) are important in initiating long‐term adaptive immunity during flu vaccination (Athale & Banchereau, 2017) with induction of plasmacytoid DCs (pDCs) that secreted type I interferons (IFNs). Young HR also showed upregulation of genes involved in B cell memory B at Day 7 (Figure S5a), which coincides with the typical appearance of antibody‐secreting cells following influenza vaccination.

### 2.4 Molecular signatures associated with response to vaccination in older subjects

**FIGURE 4:**
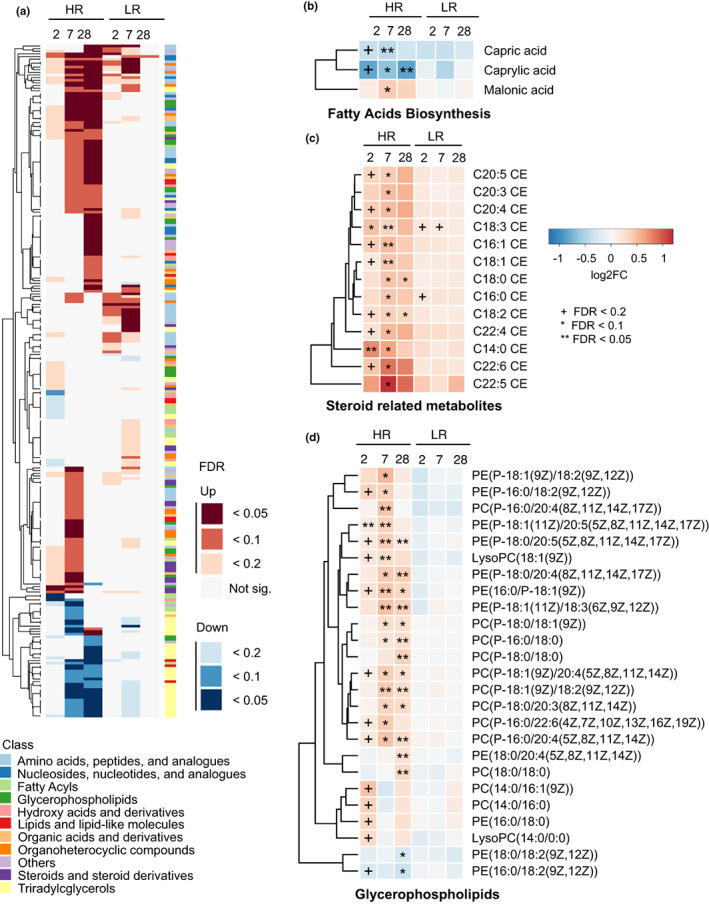
Molecular signatures associated with response to vaccination in older subjects. (a) Differentially abundant metabolite levels (absolute value of fold change ≥1.2, *p* < 0.05, and FDR < 0.2) at Day 2, Day 7, and Day 28 post‐vaccination relative to Day 0 in HR and LR groups. (b–d) Molecular signatures for the top differential metabolic pathways in old HR and LR: fatty acid biosynthesis, cholesteryl esters, and glycerophospholipids. Color labels correspond to indicated log2FC. ^+^FDR < 0.2, *FDR < 0.1, **FDR < 0.05.

Differentially abundant metabolites in older HRs and LRs were also identified at Days 2, 7, and 28 after flu vaccination by comparison with Day 0. A total of 255 DAMs were identified across all time points (Figure 4a, Table S2). Medium chain fatty acid biosynthesis (Figure 4b) and steroid‐related metabolites (Figure 4c) show similar trends in older HR vs LR groups, although changes only reached significance in HRs. Caprylic acid and capric acid are medium‐chain fatty acids that decreased after vaccination in the older HR cohort. Medium chain fatty acids are beneficial to human health as a source of energy for cells and their antibacterial and antiviral activity (Fletcher & Meredith, 2020; Huang & Tsai, 2014); however, their function in the response to influenza vaccine is unclear. Most sterol metabolites also showed similarities with an upward trend in both older HRs and LRs, but most significant changes again occur in HRs. The sterol‐related metabolites belong to the cholesteryl esters and are involved in reverse cholesterol transport. The increase in these metabolites after vaccination in the older HR group suggests that vaccination increased storage of cholesterol as cholesteryl esters. Glycerophospholipids (Figure 4d) show differing trends among responder groups, significantly increasing across post‐vaccination time points in HRs while they trend downward in LRs. The BTM profiles of old adults suggested a picture that is very different from that observed in the young cohort. Older HR had overall less induction of BTMs compared to young HR (Figure S5b); in contrast, older LR seem to promote a late NK and T cell‐based response (Figure S5b). In common with young LR, older LR individuals share a BTM signature for protein folding and phosphatidylinositol metabolism (Figure S5b), which was implicated in response to the live attenuated varicella‐zoster vaccine (Li et al., 2017).

### 2.5 Integrated metabolomic and transcriptomic signature identifies high responder signatures

**FIGURE 5:**
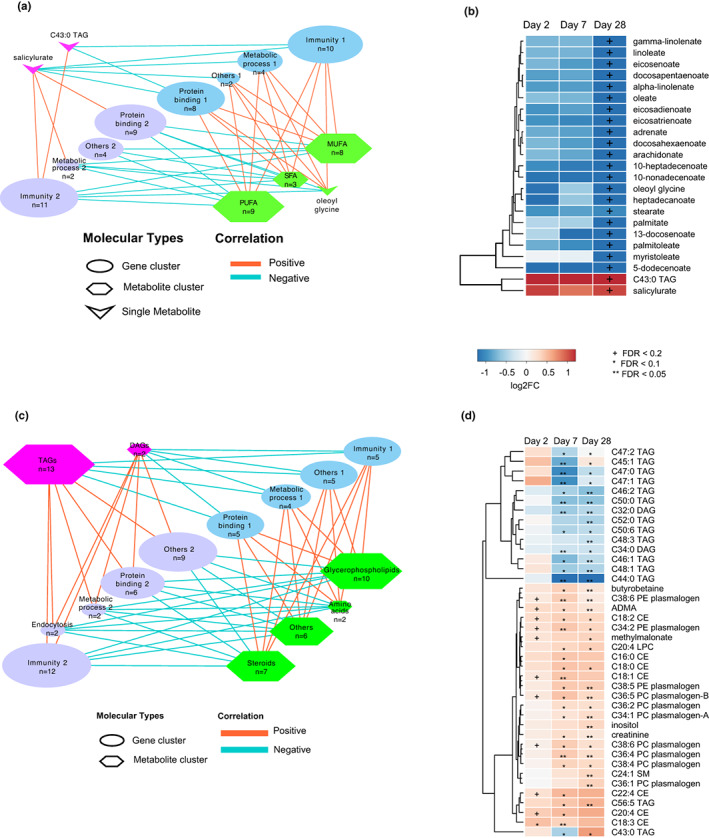
Integrated metabolomic and transcriptomic correlation and network in young and older high responders. Sparse Partial Least Squares Regression (sPLS) was used for simultaneous variable selection in the transcriptomics and metabolomics data sets in young (a, b) and older (c, d) HR. (a, c) Correlation networks of genes categorized by function and metabolites categorized by class. (b) Differentially abundant metabolites from (a). (d) Differentially abundant metabolites from (c). ^+^FDR < 0.2, *FDR < 0.1, **FDR < 0.05.

We have identified metabolites that are quantitatively differential in the older and young cohorts with high and low responder profiles to flu vaccination (Figure 3a and Figure 4a). Next, we performed sparse partial least squared (sPLS) correlation analysis of the differentially abundant metabolites in the high responder group to identify possible correlations of these metabolites with gene expression profiles found in young and older HR. We selected the results of sPLS dimension 1 with strong correlations (*R* ≥ 0.4). Figure 5a shows two different clusters of genes and metabolites in young HR, including those with inverse correlations. The metabolites identified in our analysis could be classified into four major groups: polyunsaturated fatty acids (PUFAs), monounsaturated fatty acids (MUFAs), saturated fatty acids (SFA), and others. Genes could also be classified into four major groups: immunity, protein binding, metabolic process, and others. We analyzed how these metabolites and genes correlate during the response to influenza vaccination and found that metabolites shown in the upper left part of Figure 5a increased significantly at Day 28 post‐vaccination while the metabolites shown in the lower right part of Figure 5a decreased on Day 28 post‐vaccination (Figure 5b). These differences in metabolite‐transcript correlates were also observed at Days 2 and 7 post‐vaccination, but they did not reach statistical significance. Our previous results in Figure 2e indicated that PUFAs significantly decreased in the young but not in the older cohort. In young adults, we found that PUFAs are significantly reduced at Day 28 in the high responder group, while in the low responder group, this decrease is reached early at Day 7 (Figure 3c). Furthermore, we found several genes with immune response function: CD1D, MAP3K8, EP300, LYN, MERTK, and METTL3, involved in the regulation of T‐cell activation that had a positive correlation with the abundance of PUFAs. In contrast, several genes involved in neutrophil degranulation (R‐HSA‐6798695), like BST2, CAPN1, STOM, and TMEM30A showed negative correlation with the abundance of PUFAs (Figure 5a, Figure S6a, Table S3). This suggests that broad depletion of PUFAs in HR subjects at Day 28 could be related to the immune response to influenza vaccination.

For sPLS correlation analysis in the old HR cohort (Figure 5c,d), metabolites were classified into six major groups: glycerophospholipids, steroids, TAGs, amino acids, diacylglycerols (DAGs), and others. Genes were classified into five major groups: immunity, protein binding, metabolic process, endocytosis, and others. The results show metabolites with significantly decreased abundance at Days 7 and 28 post‐vaccination in the upper left and metabolites that were significantly increased at these timepoints in the lower right panel. Six Immunity 2 group genes (MX1, OAS3, IFITM3, RSAD2, LDLR, and IFI44) were involved in interferon alpha/beta signaling (R‐HSA‐909733), response to virus (GO:0016032), and viral processes (GO:0009615), and one (KCTD12) was involved in mitotic cell cycle in stimulated CD4 T cells (M4.11) (Figure 5c, Figure S6b, Table S3). These genes showed positive correlation with TAGs/DAGs but negative correlation with glycerophospholipids and sterols. Glycerophospholipid abundance has been associated with an inflammatory phenotype (Köberlin & Snijder, 2015), and a previous study found that glycerophospholipids are increased in response to fungal infection (Wu et al., 2021). These findings may imply that the increased levels of glycerophospholipids in older HRs were associated with a strong response to flu vaccination.

## 3 DISCUSSION

We used a systems vaccinology approach to study the response to influenza vaccination and found that both young and older subjects develop an antibody response to immunization using different immunometabolic paths. Our study suggests that the main variable in response to influenza vaccination is age (Figure 1c), a finding that is in agreement with previous studies (Furman & Jojic, 2013; Haschemi & Kosma, 2012; Kennedy & Ovsyannikova, 2016; Nakaya et al., 2011; Rogers et al., 2019; Thakar et al., 2015; Tsang et al., 2014; Voigt et al., 2019). The differences we observed reflect a differential metabolic baseline of young versus older adults. Conversely, humans, like most animals in nature, are not immunologically naive. At Day 0, younger individuals had higher levels of fatty acids and fatty acid conjugates, as well as glycerophospholipids and steroid esters, when compared to older adults (Figure S2). The difference in abundance of fatty acids and PUFAs could be a common feature of aging in mammals, since a similar signature with a decrease in serum fatty acids was also observed in aging mice (Tomás‐Loba et al., 2013). In contrast, older adults had an increase in triacylglycerols and products of amino acid metabolism (Figure S2). This included C‐glycosyl‐tryptophan, a metabolite of tryptophan that strongly correlates with age (Menni et al., 2013).

Our younger and older cohorts had no significant baseline differences for serine or glycine metabolites; these amino acids are obtained from extracellular sources such as diet and are required for effector T‐cell proliferation (Ma et al., 2017). Serine metabolic genes are associated with a strong response to influenza vaccination (Tsang et al., 2014). However, older individuals showed significantly elevated levels of these amino acids after vaccination, suggesting that serine is not being consumed or is being synthesized de novo. In support of the latter view, the transcriptome signature in older individuals showed an increase in expression of serine biosynthetic enzymes (Figure S3b). Thus, the mechanisms to generate an immune response to the influenza vaccine in older cohorts may not be related to increased T‐cell proliferation as serine is accumulating instead of being consumed. Conversely, serine and glycine are limiting factors in the synthesis of glutathione, an antioxidant which is essential for regulatory T‐cell function (Kurniawan et al., 2020).

Purines have pleiotropic effects in immune cells, particularly in the activation of naive and effector T cells (Cekic & Linden, 2016) and modulation of purine metabolic genes has been correlated with response to influenza vaccine (Tsang et al., 2014). While baseline levels of purine metabolites were similar in our young and older cohorts, purine metabolism exhibited age‐specific changes after influenza immunization. Young adults had increased levels of adenosine and guanosine derivatives on Day 7, while older individuals had increased levels of hypoxanthine and xanthine, catabolic salvage pathway products of adenine and guanine. This suggests that older individuals are consuming adenine and guanine and converting them into xanthine and hypoxanthine which could be recycled to adenine and guanine through the salvage pathway. Indeed, others have found that levels of hypoxanthine and xanthine increase with age in humans (Zieliński et al., 2019). In aging athletes, there is also increased activity of the enzyme HGPRT that recycles hypoxanthine and xanthine to adenine (Zieliński et al., 2019). Conversely, older individuals may be deficient in the synthesis of purines from the de novo pathway and therefore need to use the salvage pathway to obtain sufficient adenine and guanine. Indeed, while young subjects express increased levels of PPAT and GART, the rate‐limiting enzymes for the purine biosynthetic pathway, older cohorts do not upregulate the expression of these enzymes (Figure S4a). These patterns may reflect metabolic adaptations required by older individuals to mount an effective immune response upon influenza vaccination. Furthermore, these purine intermediates may have immunologic functions of their own that shape the differentiated immune responses of old and young individuals. For example, inosine may have a pro‐inflammatory function in innate immunity as a known activator of the TLR7 and TLR8 pathway in mice (Sarvestani et al., 2014). Further research will be necessary to define the impact of purine metabolites in the response to influenza vaccines in older individuals.

We found a higher level of fatty acyl carnitines at baseline in older adults prior to influenza vaccination when compared to their younger counterparts (Table S4), which may reflect an elevated basal level of fatty acid oxidation. Notably, fatty acid oxidation is important for the generation of memory CD8^+^ T cells in mice (Pearce et al., 2009), and increased basal fatty acid oxidation is observed in CD4^+^ T cells of older individuals (>65 years) (Yanes et al., 2019). Upon immunization, the levels of fatty acyl carnitines dropped in young cohorts while increasing further in older cohorts (Figure 2f). It is possible that fatty acid oxidation is increased in younger individuals after vaccination, leading to a depletion of fatty acyl carnitines in this population. In contrast, accumulation of fatty acyl carnitines in older subjects suggests that, while the conversion of fatty acids into fatty acyl carnitines is enhanced in older individuals, their downstream catabolism is not increased at the same rate after influenza vaccination. The functional significance of these findings in older individuals is not clear.

Many triacylglycerols (TAGs) were higher at baseline (Figure S2) but reduced in older individuals after vaccination (Figure 2h), suggesting that these lipids are being depleted at a rate that is higher than their de novo synthesis. TAG synthesis is essential for T‐cell memory responses in mice (Cui & Staron, 2015) and has been associated with regulatory T‐cell function in humans (Howie & ten Bokum, 2019). In addition to potentially contributing to T‐cell memory responses, these metabolites may play a role in dampening the inflammatory response in older individuals, perhaps working in concert with the glutathione biosynthetic pathway promoting regulatory T‐cell function.

We found a modest increase of the primary bile acids cholate and taurocholate at Day 2 and Day 7 post‐vaccine in older and young individuals, respectively (Figure 2d). Young individuals showed an increase in the secondary bile acid deoxycholate at Day 7 (Figure 2d). Bile acids are catabolic products of cholesterol and can be divided between primary bile acids generated by the liver, and secondary bile acids that result from modification of primary bile acids by gut microbiota. Different types of bile acids bind to the nuclear hormone receptor FXR, either as agonists (Makishima et al., 1999; Parks et al., 1999) or as antagonists (Sayin et al., 2013). The presence of secondary and FXR antagonist bile acids was previously associated with systemic inflammation (Fu & Coulter, 2019). We also observed reduction in the levels of polyunsaturated fatty acids (PUFAs) in young compared to older individuals (Figure 2e). These fatty acids are precursors for many immunomodulatory molecules with both pro‐ and anti‐inflammatory activities like prostaglandins, leukotrienes, resolvins, and maresins (Serhan, 2014; Serhan & Petasis, 2011). In this sense, it is interesting that in HRs there is an enrichment of sterol esters, conjugates of mainly cholesterol and fatty acids, that are composed of PUFAs compared to more saturated fatty acids (Figure 4c). These sterol esters may be used as storage for PUFAs that can be used to mount a robust immune response to influenza vaccine.

Transcriptomic analyses suggest that myeloid signatures are largely suppressed in young and older subjects after vaccination (Figure S4c). Older subjects tend to have higher signatures for NK cells (Nakaya et al., 2015), accumulate mature active CD56^dim^ CD16^+^ NK cells (Solana et al., 2014), and show a reduced signature for type I interferon responses (Figure S4c). The proinflammatory gene expression profile starts to be downregulated at Days 7 to 28 post‐vaccination (Figure S4c). In contrast, the phosphatidylinositol signature increases in young subjects, reaching a peak at Day 28, while older cohorts start from a higher level at Day 2 but this is decreased at later time points (Figure S4c). In BTM analysis, enriched in T cells (I) (M7.0) is upregulated at Day 7 in older subjects. The T‐cell activation (II) (M7.3) module is only upregulated in young subjects at Day 28 post‐vaccination, while in older subjects it is upregulated early on (Day 2) (Figure S4c). Previous studies showed that an increase in phosphatidylinositol metabolism after vaccination correlates with high T‐cell response to the shingles Zostavax vaccine (Li et al., 2017). Our findings suggest that the phosphatidylinositol metabolism module is upregulated early on after flu vaccination in older adults and more specifically on those in the high responder group (Figure S5b). This correlates well with the early upregulation in older adults of the T‐cell activation module at Day 2 and the signaling in T cells and enriched in T‐cell modules at Day 7 (Figure S4c).

Comparison of HR and LR in the young and older adults suggests that older HRs have a subtle and early response to vaccination that is at the transcriptomic level nearly undetectable in our sample (Figure S5b). This contrasts with both young HR that show an immune response driven by strong antigen presentation and IFN response (Figure S5a). Glycerophospholipids are widely distributed in biological membranes and may play a role in immune responses (O'Donnell et al., 2018). Our study found changes in levels of phosphatidylethanolamines (PEs) and phosphatidylcholines (PCs) in early timepoints of the immune response to flu vaccine in older HRs (Figure 4d). PE is important in signaling and metabolic pathways that stimulate T‐cell activation (Ma et al., 2021), and alterations in PE and PC levels have been observed in autoimmune diseases (Mendes‐Frias et al., 2020; Zeng et al., 2017). Increase in both PC and PE was associated with differentiation of naive T cells into T_FH_ cells (Fu & Guy, 2021) and may impact robustness of vaccine response (Deng & Chen, 2021; Koutsakos & Wheatley, 2018).

In contrast to the young cohort, in older patients the decline in immune response with age results in impaired effector T‐cell development, functionality, and long‐term memory generation (Gustafson & Kim, 2020). Notably, we found that older LRs tend to promote late NK and T cell‐based responses, while young LRs seem to have a problem in building a response to the vaccine. Thus, our study suggests that older subjects may rely more on memory or cross‐memory responses than younger adults, who seem to rely more on immune responses driven by antigen presentation and IFN signaling. This is consistent with previous studies describing elevated memory T cells (Furman & Jojic, 2013) and expansion of atypical memory B cells (CD10^−^CD20^+^CD21^−^CD27^−^) and age‐associated B cells (ABC, CD21^−^T‐bet^+^CD11c^+^) in older populations (Nipper et al., 2018). It is possible that most older adults in the LR group may still develop a strong memory component but this may occur at a later time point than Day 28 post‐vaccine. Future studies are needed to clarify whether older LRs form a delayed memory response and whether they would benefit from vaccine boosters or higher dosages. Furthermore, there is a need to identify the underlying causes of LR in young adults. These differences could be explored to increase the potency of vaccines for young and old subjects.

Young HRs show high correlation of immunomodulatory PUFAs (Serhan, 2014; Serhan & Petasis, 2011) and genes involved in the regulation of T‐cell responses. Unsaturated fatty acids are precursors of prostaglandins and eicosanoids and have known immunomodulatory properties (Serhan, 2014; Serhan & Petasis, 2011), including stimulation of IL‐1α (Freigang & Ampenberger, 2013) and involvement in T‐cell immunity (Nicolaou et al., 2014); therefore, it is possible that the broad depletion of PUFAs observed in HR subjects is caused by and contributes to a robust immune response to vaccination. Furthermore, in young LR, PUFAs are quickly consumed and most likely are not available in enough concentrations to generate such a strong response. Indeed, high dietary intake of PUFAs can affect the immune response to vaccines in mice (Hogenkamp & van Vlies, 2011) and arachidonic acid, a PUFA, can increase response to flu vaccine in humans (Kelley & Taylor, 1997). Further studies are needed to determine whether increasing PUFAs in the diet of young individuals can contribute to higher response rates to flu vaccine. In contrast, older HRs showed a strong correlation with genes that are involved in rapid virus clearance, repression of the IFN response and positive regulation of the CD4 T‐cell response with the presence of TAG/DAGs; in this regard, TAGs are associated with CD8+ T‐cell memory in mice (Cui & Staron, 2015).

Taken together, the metabolomic and transcriptomic signature after vaccination with influenza shows that young subjects rely on strong T‐ and B‐cell activation, which is supported by our previous transcriptomic analysis of influenza vaccine response (HIPC‐CHI Signatures Project Team and HIPC‐I Consortium, 2017). Accordingly, young groups show increased levels of adenosine and guanine, phosphatidylinositol metabolism and reduced levels of fatty acyl carnitines, suggesting increased fatty acid oxidation. Younger cohorts show higher plasma levels of secondary and FXR antagonistic bile acids, an indication of possible systemic inflammatory response. In contrast, older cohorts accumulate amino acids serine and glycine which are involved in glutathione metabolism essential for regulatory T‐cell function. Older subjects had lower increases over time in phosphatidylinositol metabolism and elevated consumption of TAGs, which in humans has been associated with regulatory T‐cell function (Howie & ten Bokum, 2019). Thus, older individuals may rely more on T‐cell memory and regulatory T cells for effective responses to influenza vaccinations. Future trials will benefit from targeting young and older cohorts differently in influenza vaccine studies.

### 3.1 Limitations of this study

Although the cohort used in this study is well characterized and was used to successfully establish associations between gene signatures and flu vaccine response, our study has several limitations. One limitation is the limited subject sample size, which may reduce our ability to establish strong correlations between molecular signatures and response to flu vaccination. Furthermore, sample size also limited our ability to detect several metabolites that have small, but significant, contributions to the response to flu vaccine. Future studies with larger sample size would increase our chances to identify less robust, but potentially biologically relevant, gene and metabolite signatures using more strict statistical tests. Another limitation is that a considerable number of features identified in our untargeted metabolomics approach are chemically unidentified, and therefore, our analysis is limited to known, well‐annotated metabolites. Future studies will be required to identify molecular signatures originating from these unknown metabolites, to define their structures and identify biologically meaningful metabolite‐gene associations. These studies can only be achieved using well‐characterized cohorts of vaccinated patients, such as the one we describe in this study.

## 4 METHODS

### 4.1 Sample collection and preparation

#### 4.1.1 Plasma sample isolation

A cohort comprised of 33 individuals (16 young individuals, age 21–30) and 17 older adults (age ≥65 years) was studied. These individuals were recruited and studied at the gene expression level in consecutive influenza vaccine seasons (2010–2011 and 2011–2012) in which the composition of the influenza vaccine was identical (Thakar et al., 2015). Participants were consented under a research protocol approved by the Human Subjects Research Protection Program of the Yale School of Medicine. Participants with an acute illness 2 weeks prior to recruitment were excluded from the study, as were individuals with primary or acquired immune‐deficiency, use of immunomodulating medications including steroids or chemotherapy, a history of malignancy other than localized skin or prostate cancer, or a history of cirrhosis or renal failure requiring hemodialysis. Whole blood was collected on pre‐vaccination Day 0 and post‐vaccination Day 2, Day 7, and Day 28 into EDTA lavender top tubes and plasma supernatant without cell debris were stored at −80°C until further use.

#### 4.1.2 HAI titer measurement and response end point definition

Hemagglutination inhibition assays were performed as previously described (Thakar et al., 2015). maxRBA an automated metric that adjusts for inverse correlations between HAI titer fold changes and baseline titers was used to classify high responders (HR) and low responders (LR) to vaccination as previously described (Avey & Mohanty, 2020). Briefly, young and older cohorts were separated, and endpoints were calculated in each season and each age group separately. Baseline and fold changes were log_2_ transformed, and an exponential curve was fit to the fold change versus baseline titers for each strain. Next, the residuals were calculated, and for each subject, the maximum residual across all strains was selected as the maxRBA. Finally, HR and LR were defined as the top and bottom 40th percentile of maxRBA, respectively. A total of 13 HR and 16 LR (6 HR and 8 LR in young subjects and 7 HR and 8 LR in older subjects) and 4 with an indeterminate response were classified using this approach. The code to calculate maxRBA is available in the *Calculate_maxRBA()* function from the titer R package (https://bitbucket.org/kleinstein/titer).

#### 4.1.3 LC–MS untargeted metabolomics profiling from plasma samples

The plasma metabolomic profiles of participants were measured from plasma samples using a combination of four LC–MS methods that measure complementary metabolites: two methods that measure polar metabolites, a method that measures metabolites of intermediate polarity (e.g., fatty acids and bile acids), and a lipid profiling method (see below for method‐specific details). For the analysis queue in each method, participants were randomized and longitudinal samples from each participant were randomized and analyzed as a group. As the aliquots for the LC–MS methods were prepared from each sample, a pooled sample was created by combining an additional aliquot from each sample into a 50 ml conical centrifuge tube. The pooled sample was mixed by vortexing and sub‐aliquoted to create pooled plasma QC samples, which were injected in pairs at intervals of approximately 20 samples for QC and data standardization.

Samples were prepared for each method using extraction procedures that are matched for use with the chromatography conditions. Data were acquired using LC–MS systems consisting of Nexera X2 U‐HPLC systems (Shimadzu Scientific Instruments) coupled to Q Exactive/Exactive Plus orbitrap mass spectrometers (Thermo Fisher Scientific).

##### LC–MS Method 1 – HILIC‐pos

Positive ion mode MS analyses of polar metabolites. LC–MS samples were prepared from or plasma (10 μl) via protein precipitation with the addition of nine volumes (90 μl) of 74.9:24.9:0.2 v/v/v acetonitrile/methanol/formic acid containing stable isotope‐labeled internal standards (valine‐d8, Isotec; and phenylalanine‐d8, Cambridge Isotope Laboratories). The samples were centrifuged (10 min, 9000 g, 4°C), and the supernatants injected directly onto a 150 × 2 mm Atlantis HILIC column (Waters). The column was eluted isocratically at a flow rate of 250 μl/min with 5% mobile phase A (10 mM ammonium formate and 0.1% formic acid in water) for 1 min followed by a linear gradient to 40% mobile phase B (acetonitrile with 0.1% formic acid) over 10 min. The column was kept at 30°C. MS analyses were carried out using electrospray ionization in the positive ion mode using full scan analysis over *m*/*z* 70–800 at 70,000 resolution and 3 Hz data acquisition rate. Additional MS settings are as follows: ion spray voltage, 3.5 kV; capillary temperature, 350°C; probe heater temperature, 300°C; sheath gas, 40; auxiliary gas, 15; and S‐lens RF level 40.

##### LC–MS Method 2 – HILIC‐neg

Negative ion mode MS analysis of polar metabolites. LC–MS samples were prepared from plasma (30 μl) via protein precipitation with the addition of four volumes (120 μl) of 80% methanol containing inosine‐15N4, thymine‐d4, and glycocholate‐d4 internal standards (Cambridge Isotope Laboratories). The samples were centrifuged (10 min, 9000 g, 4°C), and the supernatants were injected directly onto a 150 × 2.0 mm Luna NH2 column (Phenomenex). The column was eluted at a flow rate of 400 μl/min with initial conditions of 10% mobile phase A (20 mM ammonium acetate and 20 mM ammonium hydroxide in water) and 90% mobile phase B (10 mM ammonium hydroxide in 75:25 v/v acetonitrile/methanol) followed by a 10 min linear gradient to 100% mobile phase A. The column temperature was kept at 40°C. MS analyses were carried out using electrospray ionization in the negative ion mode using full scan analysis over *m*/*z* 70–750 at 70,000 resolution and 3 Hz data acquisition rate. Additional MS settings are as follows: ion spray voltage, −3.0 kV; capillary temperature, 350°C; probe heater temperature, 325°C; sheath gas, 55; auxiliary gas, 10; and S‐lens RF level 50.

##### LC–MS Method 3 – C18‐neg

Negative ion mode analysis of metabolites of intermediate polarity (e.g., bile acids and free fatty acids). Plasma (30 μl) was extracted using 90 μl of methanol containing PGE2‐d4 as an internal standard (Cayman Chemical Co.) and centrifuged (10 min, 9000 g, 4°C). The supernatants (10 μl) were injected onto a 150 × 2.1 mm ACQUITY BEH C18 column (Waters). The column was eluted isocratically at a flow rate of 450 μl/min with 20% mobile phase A (0.01% formic acid in water) for 3 min followed by a linear gradient to 100% mobile phase B (0.01% acetic acid in acetonitrile) over 12 min. The column temperature was kept at 45°C. MS analyses were carried out using electrospray ionization in the negative ion mode using full scan analysis over *m*/*z* 70–850 at 70,000 resolution and 3 Hz data acquisition rate. Additional MS settings are as follows: ion spray voltage, −3.5 kV; capillary temperature, 320°C; probe heater temperature, 300°C; sheath gas, 45; auxiliary gas, 10; and S‐lens RF level 60.

##### LC–MS Method 4 – C8‐pos

Lipids (polar and nonpolar) were extracted from plasma (10 μl) using 190 μl of isopropanol containing 1‐dodecanoyl‐2‐tridecanoyl‐sn‐glycero‐3‐phosphocholine as an internal standard (Avanti Polar Lipids). After centrifugation (10 min, 9000 g, ambient temperature), supernatants (10 μl) were injected directly onto a 100 × 2.1 mm ACQUITY BEH C8 column (1.7 μm; Waters). The column was eluted at a flow rate of 450 μl/min isocratically for 1 minute at 80% mobile phase A (95:5:0.1 vol/vol/vol 10 mM ammonium acetate/methanol/acetic acid), followed by a linear gradient to 80% mobile‐phase B (99.9:0.1 vol/vol methanol/acetic acid) over 2 min, a linear gradient to 100% mobile phase B over 7 min, and then 3 min at 100% mobile phase B. The column temperature was kept at 30°C. MS analyses were carried out using electrospray ionization in the positive ion mode using full scan analysis over *m*/*z* 200–1100 at 70,000 resolution and 3 Hz data acquisition rate. Additional MS settings are as follows: ion spray voltage, 3.0 kV; capillary temperature, 300°C; probe heater temperature, 300°C; sheath gas, 50; auxiliary gas, 15; and S‐lens RF level 60.

##### Data processing

Nontargeted data were processed using Progenesis QI software (v 2.0, Nonlinear Dynamics) to detect and de‐isotope peaks, perform chromatographic retention time alignment, and integrate peak areas. Identification of nontargeted metabolite LC–MS peaks were conducted by matching measured retention times (RT) and mass to charge ratios (*m*/*z*) to mixtures of reference metabolites analyzed in each batch. Additionally, we matched unknown features in the data set to an internal database of >600 compounds that have been characterized using the Broad Institute methods. This library contains compounds that have been confirmed by matching their RT, *m*/*z*, and MS/MS fragmentation patterns in multiple human biofluids in previous studies using authentic reference standards. To annotate unknowns in this dataset using this library, we used in‐house alignment scripts to adjust the RT and *m*/*z* and match study unknowns to the compound library. No MS/MS was generated for this study. Temporal drift was monitored and normalized with the intensities of features measured in one of the doubly injected QC pooled reference samples using a nearest neighbor approach, where sample intensities in each QC pool are used to scale their closest samples in the batch. To determine the analytical precision of the method for each measured metabolite, we computed coefficients of variation (CV) for annotated and unknown features using the remaining QC pools not used for scaling temporal drifts. The average CV values per method for annotated compounds ranged from 7% to 11%, which is within the historical analytical precision of the methods applied. Finally, principal component analyses were generated and scores plots used to determine the presence of any potential outlying samples.

### 4.2 Metabolomics computational and statistical analysis

The relative intensities of metabolites were pre‐processed, normalized, and log‐transformed for further analysis. Metabolites that were not detected (NA) in more than 50% of samples were removed, and the remaining NA metabolites were imputed with half of the minimum value of that metabolite. The metabolites were median normalized within samples, and their intensities were scaled by multiplying by 10^6^ and log‐transforming to stabilize variance. To investigate how factors such as age, gender, response, and time point contributed to the variation of metabolomics profiles, we used *lme4*, an R package for linear mixed models, to calculate means of the F value of each factor (fixed effects) and the same subjects as random effects (log_2_(normalized metabolite) ~ AgeGroup + Gender + Response + TimePoint + (1|subject)). We also used principal component analyses to characterize each group of factors. To identify differentially abundant metabolites pre‐ (Day 0) and post‐vaccination (Days 2, 7, and 28) in young and older subjects, we used a *lme4* to fit a linear mixed model with the time point as fixed effect and the same subjects as random effects (log_2_(normalized metabolite) ~ TimePoint + (1|subject)). Linear mixed models were applied on normalized log_2_‐transformed data. One‐way ANOVA testing was used to evaluate *p*‐values at each time point (Days 2, 7, and 28) relative to baseline (Day 0). *p*‐Values were corrected for multiple comparisons using the Storey method (Storey & Tibshirani, 2003) to calculate false discovery rate (FDR). Differentially abundant metabolites (DAM) were defined by thresholds of *p‐*value <0.05 and |FC| ≥ 1.2 with results displayed for FDR of <0.05, <0.1, and <0.2 in figures, as indicated. Hierarchical clustering and heatmap generation were performed by Morpheus (https://software.broadinstitute.org/morpheus) based on Euclidean distance or by R using heatmap.3 library. To identify similar and closely related metabolites, we use human metabolome database (HMDB) (Wishart et al., 2018) to classify the metabolites into the same subclass or pathways.

### 4.3 Transcriptomics samples and computational and statistical analysis

Transcriptomics data from the same cohort were downloaded from GSE59654. We selected data from the same 33 subjects matching those for which we obtained metabolomics data. Transcriptomic data were available for all 33 subjects at Day 0 and Day 7, while for Days 2 and 28 data were available for 29 and 31 subjects, respectively. Differentially expressed genes (DEGs) across different time points were determined using the limma package with GEO2R in R. DEGs were defined by thresholds of *p*‐value <0.05 and FC ≥ 1.25 or ≤ 1/1.25. To identify similar gene signatures from PBMC transcriptomics data, we performed Blood Transcription Modules analysis by BTM tools (Li et al., 2014). Fisher's exact test was performed on the differentially expressed gene lists for each BTM. ‐logp‐values were used in the heatmap, where positive values represent modules enriched among the upregulated genes and the negative represent modules enriched in the downregulated genes. Hierarchical clustering and heatmap generation were performed by Morpheus based on Euclidean distance between ‐logP vectors.

### 4.4 Integrated analysis of metabolomics and transcriptomics

Sparse partial least squared was performed using mixOmics (Rohart et al., 2017) to identify highly positively and negatively correlated genes and metabolites. Matching samples from the gene and metabolic profiles were first identified. Because the transcriptomic data for Days 2 and 28 were only available for 29 and 31 subjects, respectively, the metabolic datasets also need to remove 4 and 2 unmatched subjects, respectively, to obtain a complete set of matched samples. We selected the differentially abundant metabolites in young and old high responder groups to explore their highly correlated genes. We selected the top 5000 variable genes from their normalized profiles. Finally, these genes and metabolites were then fed into sPLS in the R package mixOmics to identify highly positive and negative correlations. The clusters of gene sets from sPLS were used to identify gene functions using Metascape (Zhou et al., 2019). The correlation networks were visualized using Cytoscape (Shannon et al., 2003) v3.8.2.

## AUTHOR CONTRIBUTION

A.C.S. and R.J.X. conceived and designed the study. C.‐H.C. designed and performed the analysis. L.K. assisted with analysis and helped supervise the project. C.‐H.C., F.R.S., and H.K. wrote the manuscript. J.A.‐P., K.P., S.J., K.B., and C.C. generated metabolomics data. S.R.J., I.U., L.D., K.R., and S.M. collected and processed clinical samples.

## CONFLICT OF INTEREST

R.J.X. is a co‐founder of Celsius Therapeutics and Jnana Therapeutics.

## Supporting information


Figure S1–S6

Table S1

Table S2

Table S3

Table S4

## Data Availability

Metabolomics data are available at ImmPort (immport.org) under accession number SDY1968.
