# Supplementary material for: Metabolomic and transcriptomic signatures of influenza vaccine response in healthy young and older adults
Source: Aging Cell. 2022 Aug 23;21(9):e13682. doi: 10.1111/acel.13682 (PMC9470889; doi:10.1111/acel.13682)
Supplement: Supplementary file 1 — Figure S1–S6 [file ACEL-21-e13682-s005.pdf]

Figure S1

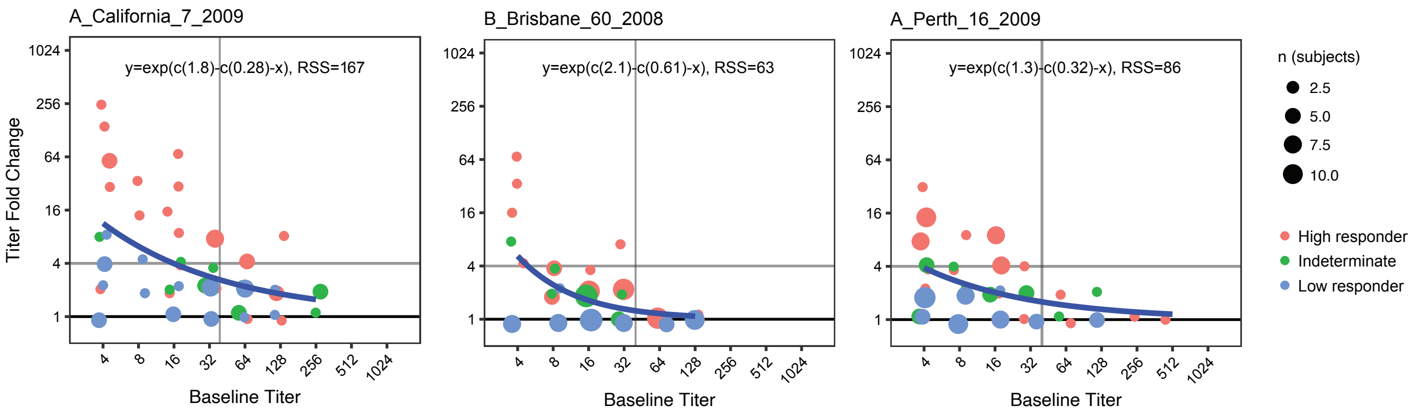

A_California_7_2009
y=exp(c(1.8)-c(0.28)-x), RSS=167
B_Brisbane_60_2008
y=exp(c(2.1)-c(0.61)-x), RSS=63
A_Perth_16_2009
y=exp(c(1.3)-c(0.32)-x), RSS=86
Titer Fold Change
1024
256
64
16
4
1
4
8
16
32
64
128
256
512
1024
Baseline Titer
n (subjects)
2.5
5.0
7.5
10.0
High responder
Indeterminate
Low responder

Figure S1. Bubble charts of pre-vaccination HAI titer versus titer fold change post-vaccination. An exponential curve was used to fit the fold-change versus baseline titers for each indicated vaccine strain. Subjects for which fold-change ≥ 1 are shown. The maxRBA was calculated using the maximum residual across all vaccine strains in the combined young and older adult cohorts to allow for comparison across age groups. The final maxRBA was then used to bin each individual into HR or LR categories.

Figure S2

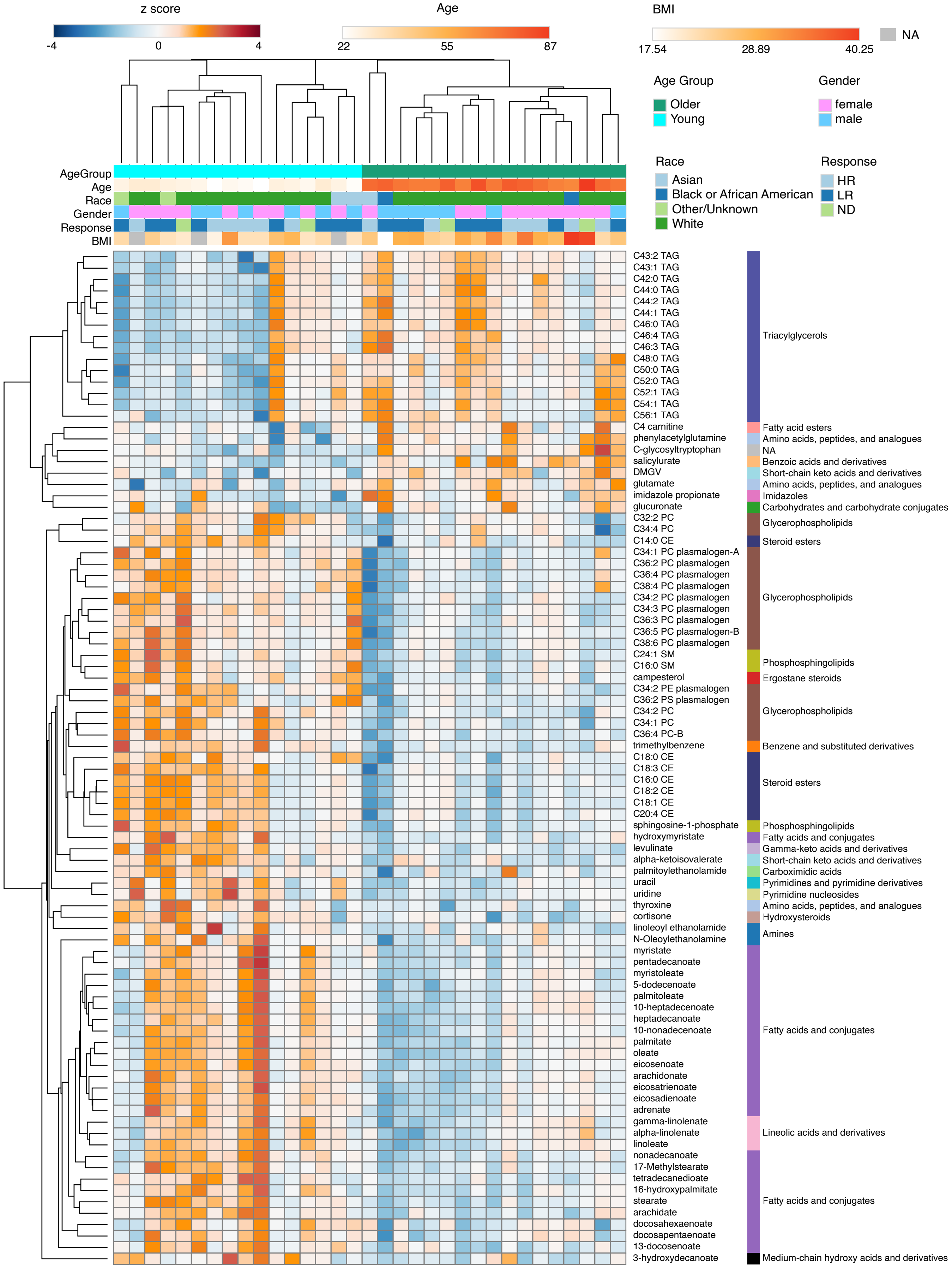
z score
-4
0
4
Age
22
55
87
BMI
17.54
28.89
40.25
NA
Age Group
Older
Young
Gender
female
male
Race
Asian
Black or African American
Other/Unknown
White
Response
HR
LR
ND
AgeGroup
Age
Race
Gender
Response
BMI
C43:2 TAG
C43:1 TAG
C42:0 TAG
C44:0 TAG
C44:2 TAG
C44:1 TAG
C46:0 TAG
C46:4 TAG
C46:3 TAG
C48:0 TAG
C50:0 TAG
C52:0 TAG
C52:1 TAG
C54:1 TAG
C56:1 TAG
C4 carnitine
phenylacetylglutamine
C-glycosyltryptophan
salicylurate
DMGV
glutamate
imidazole propionate
glucuronate
C32:2 PC
C34:4 PC
C14:0 CE
C34:1 PC plasmalogen-A
C36:2 PC plasmalogen
C36:4 PC plasmalogen
C38:4 PC plasmalogen
C34:2 PC plasmalogen
C34:3 PC plasmalogen
C36:3 PC plasmalogen
C36:5 PC plasmalogen-B
C38:6 PC plasmalogen
C24:1 SM
C16:0 SM
campesterol
C34:2 PE plasmalogen
C36:2 PS plasmalogen
C34:2 PC
C34:1 PC
C36:4 PC-B
trimethylbenzene
C18:0 CE
C18:3 CE
C16:0 CE
C18:2 CE
C18:1 CE
C20:4 CE
sphingosine-1-phosphate
hydroxymyristate
levulinate
alpha-ketoisovalerate
palmitoylethanolamide
uracil
uridine
thyroxine
cortisone
linoleoyl ethanolamide
N-Oleoylethanolamine
myristate
pentadecanoate
myristoleate
5-dodecenoate
palmitoleate
10-heptadecenoate
heptadecanoate
10-nonadecenoate
palmitate
oleate
eicosenoate
arachidonate
eicosatrienoate
eicosadienoate
adrenate
gamma-linolenate
alpha-linolenate
linoleate
nonadecanoate
17-Methylstearate
tetradecanedioate
16-hydroxypalmitate
stearate
arachidate
docosahexaenoate
docosapentaenoate
13-docosenoate
3-hydroxydecanoate
Triacylglycerols
Fatty acid esters
Amino acids, peptides, and analogues
NA
Benzoic acids and derivatives
Short-chain keto acids and derivatives
Amino acids, peptides, and analogues
Imidazoles
Carbohydrates and carbohydrate conjugates
Glycerophospholipids
Steroid esters
Glycerophospholipids
Phosphosphingolipids
Ergostane steroids
Glycerophospholipids
Benzene and substituted derivatives
Steroid esters
Phosphosphingolipids
Fatty acids and conjugates
Gamma-keto acids and derivatives
Short-chain keto acids and derivatives
Carboximidic acids
Pyrimidines and pyrimidine derivatives
Pyrimidine nucleosides
Amino acids, peptides, and analogues
Hydroxysteroids
Amines
Fatty acids and conjugates
Lineolic acids and derivatives
Fatty acids and conjugates
Medium-chain hydroxy acids and derivatives

Figure S2. Heatmap of differentially abundant metabolites at day 0 in young and old.

Differentially abundant metabolite levels (absolute value of fold-change $\geq$ 1.2 and $p < 0.05$) prior to vaccination in young and older groups. Characteristics of participants (age, race, gender, vaccine response, and BMI) are indicated. Subjects that were not classified as HR or LR (ND) were omitted from response-related analyses. Lines arranged vertically on the left group metabolites that cluster similarly and lines across the top indicate similar clustering of subjects within each age group.

Figure S3

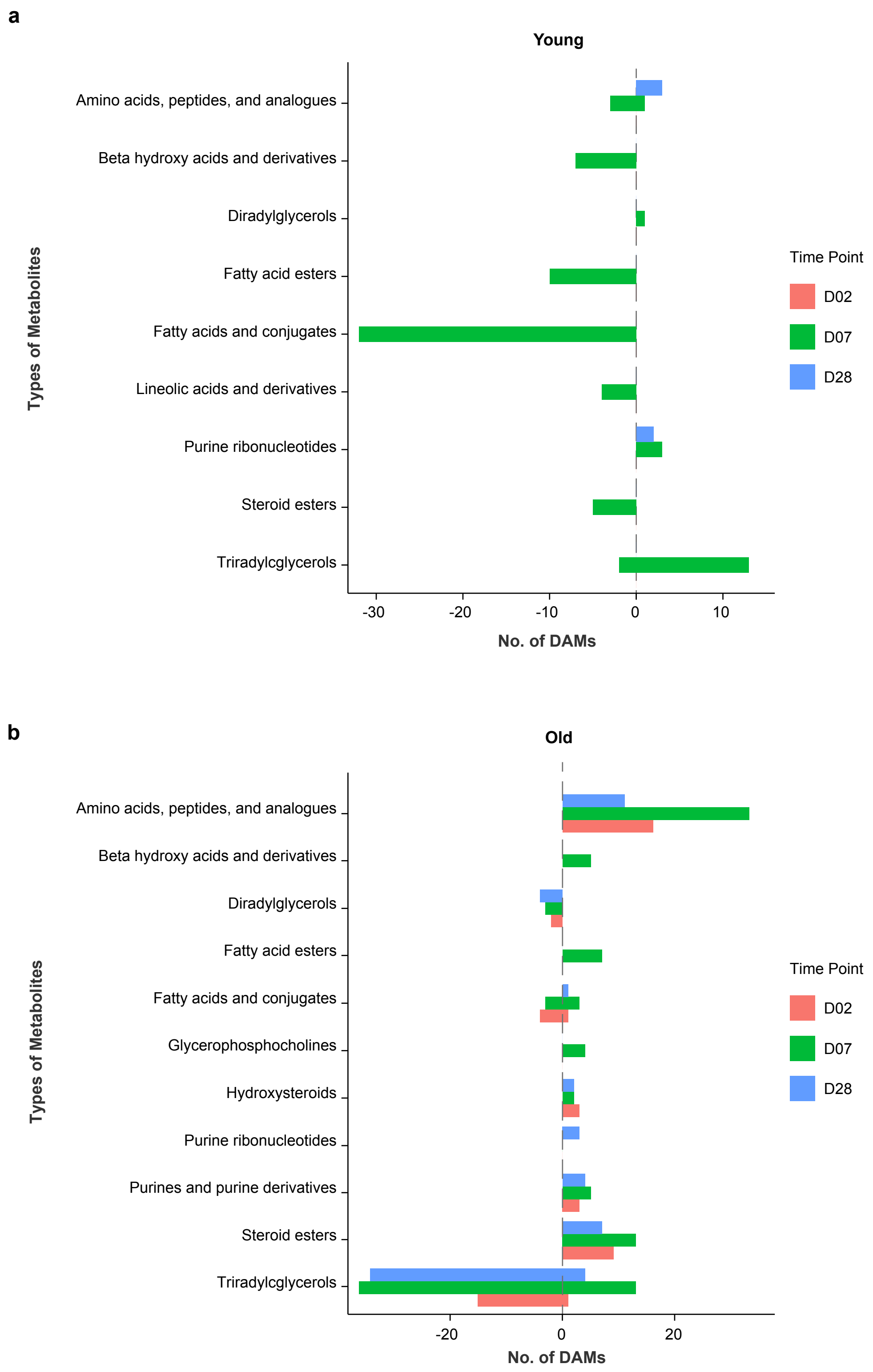
a
Young
Amino acids, peptides, and analogues
Beta hydroxy acids and derivatives
Diradylglycerols
Fatty acid esters
Fatty acids and conjugates
Lineolic acids and derivatives
Purine ribonucleotides
Steroid esters
Triradylcglycerols
Types of Metabolites
-30
-20
-10
0
10
No. of DAMs
Time Point
D02
D07
D28
b
Old
Amino acids, peptides, and analogues
Beta hydroxy acids and derivatives
Diradylglycerols
Fatty acid esters
Fatty acids and conjugates
Glycerophosphocholines
Hydroxysteroids
Purine ribonucleotides
Purines and purine derivatives
Steroid esters
Triradylcglycerols
Types of Metabolites
-20
0
20
No. of DAMs
Time Point
D02
D07
D28

Figure S3. Overview of differentially abundant metabolites (DAMs) after vaccination by metabolite class in young and older adults. Differentially abundant metabolite levels (absolute value of fold-change ≥ 1.2 and $p < 0.05$) at day 2, 7, and 28 post-vaccination relative to day 0 in young and older groups.

Figure S4

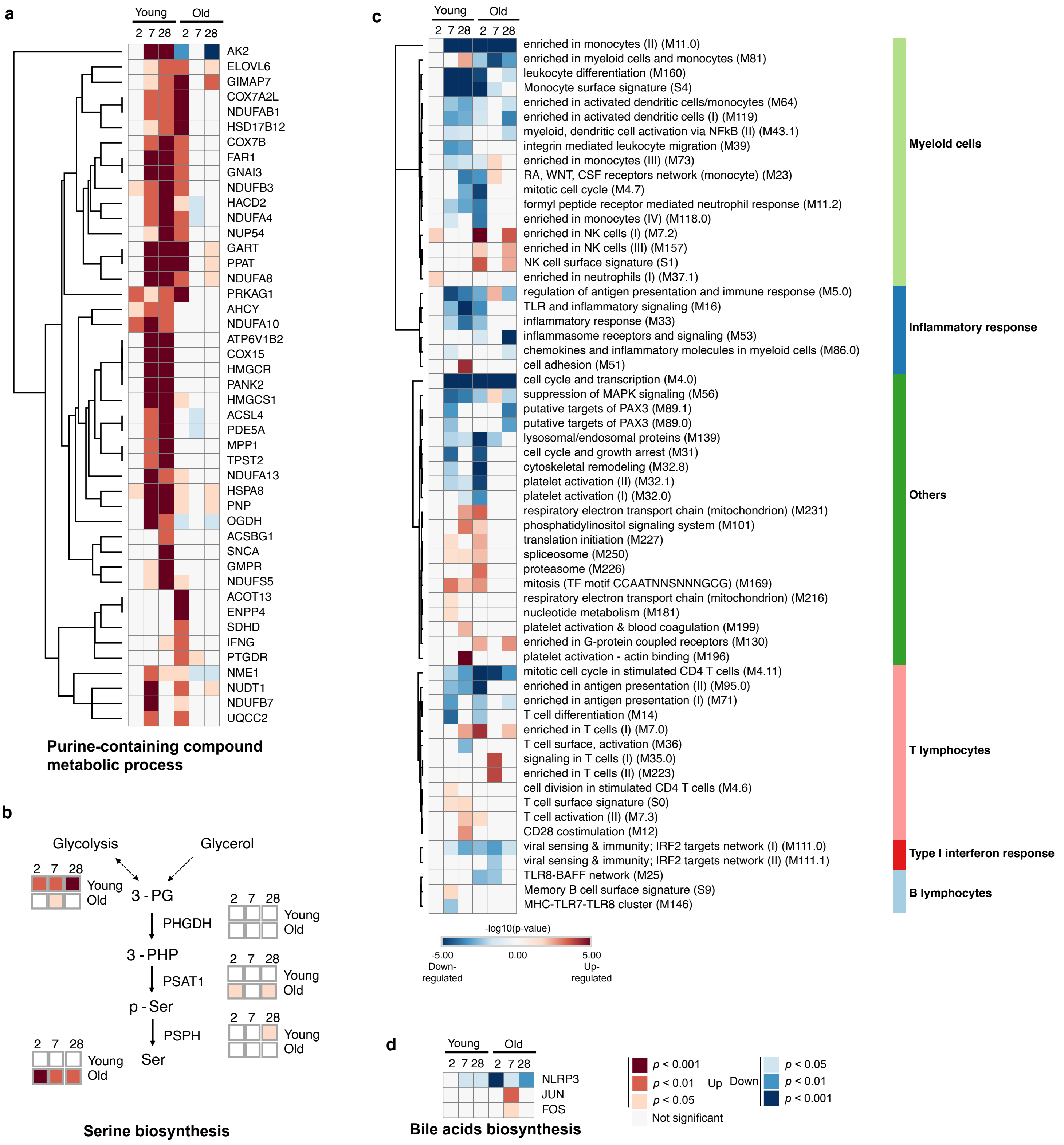
a
Young
Old
2 7 28 2 7 28
AK2
ELOVL6
GIMAP7
COX7A2L
NDUFAB1
HSD17B12
COX7B
FAR1
GNAI3
NDUFB3
HACD2
NDUFA4
NUP54
GART
PPAT
NDUFA8
PRKAG1
AHCY
NDUFA10
ATP6V1B2
COX15
HMGCR
PANK2
HMGCS1
ACSL4
PDE5A
MPP1
TPST2
NDUFA13
HSPA8
PNP
OGDH
ACSBG1
SNCA
GMPR
NDUFS5
ACOT13
ENPP4
SDHD
IFNG
PTGDR
NME1
NUDT1
NDUFB7
UQCC2
Purine-containing compound metabolic process
b
Glycolysis
Glycerol
2 7 28
Young
Old
3 - PG
PHGDH
3 - PHP
PSAT1
p - Ser
PSPH
Ser
Serine biosynthesis
c
Young
Old
2 7 28 2 7 28
enriched in monocytes (II) (M11.0)
enriched in myeloid cells and monocytes (M81)
leukocyte differentiation (M160)
Monocyte surface signature (S4)
enriched in activated dendritic cells/monocytes (M64)
enriched in activated dendritic cells (I) (M119)
myeloid, dendritic cell activation via NFkB (II) (M43.1)
integrin mediated leukocyte migration (M39)
enriched in monocytes (III) (M73)
RA, WNT, CSF receptors network (monocyte) (M23)
mitotic cell cycle (M4.7)
formyl peptide receptor mediated neutrophil response (M11.2)
enriched in monocytes (IV) (M118.0)
enriched in NK cells (I) (M7.2)
enriched in NK cells (III) (M157)
NK cell surface signature (S1)
enriched in neutrophils (I) (M37.1)
regulation of antigen presentation and immune response (M5.0)
TLR and inflammatory signaling (M16)
inflammatory response (M33)
inflammasome receptors and signaling (M53)
chemokines and inflammatory molecules in myeloid cells (M86.0)
cell adhesion (M51)
cell cycle and transcription (M4.0)
suppression of MAPK signaling (M56)
putative targets of PAX3 (M89.1)
putative targets of PAX3 (M89.0)
lysosomal/endosomal proteins (M139)
cell cycle and growth arrest (M31)
cytoskeletal remodeling (M32.8)
platelet activation (II) (M32.1)
platelet activation (I) (M32.0)
respiratory electron transport chain (mitochondrion) (M231)
phosphatidylinositol signaling system (M101)
translation initiation (M227)
spliceosome (M250)
proteasome (M226)
mitosis (TF motif CCAATNNSNNNGCG) (M169)
respiratory electron transport chain (mitochondrion) (M216)
nucleotide metabolism (M181)
platelet activation & blood coagulation (M199)
enriched in G-protein coupled receptors (M130)
platelet activation - actin binding (M196)
mitotic cell cycle in stimulated CD4 T cells (M4.11)
enriched in antigen presentation (II) (M95.0)
enriched in antigen presentation (I) (M71)
T cell differentiation (M14)
enriched in T cells (I) (M7.0)
T cell surface, activation (M36)
signaling in T cells (I) (M35.0)
enriched in T cells (II) (M223)
cell division in stimulated CD4 T cells (M4.6)
T cell surface signature (S0)
T cell activation (II) (M7.3)
CD28 costimulation (M12)
viral sensing & immunity; IRF2 targets network (I) (M111.0)
viral sensing & immunity; IRF2 targets network (II) (M111.1)
TLR8-BAFF network (M25)
Memory B cell surface signature (S9)
MHC-TLR7-TLR8 cluster (M146)
Myeloid cells
Inflammatory response
Others
T lymphocytes
Type I interferon response
B lymphocytes
-log10(p-value)
-5.00 Down-regulated
0.00
5.00 Up-regulated
d
Young
Old
2 7 28 2 7 28
NLRP3
JUN
FOS
Bile acids biosynthesis
p < 0.001
p < 0.01
p < 0.05
Up
Down
p < 0.05
p < 0.01
p < 0.001
Not significant

Figure S4. Differential molecular signatures induced by flu vaccination in young and older cohorts. For transcriptomic data, n=33 for days 0 and 7, n=29 for day 2, and n=31 for day 28. (**a**) The gene expression signature in purine-derived metabolic processes (GO:0072521). (**b**) Gene and metabolite signature in the serine biosynthesis pathway. (**c**) Blood transcription module (BTM) signatures. (**d**) Selected differentially expressed genes involved in bile acid biosynthesis.

Figure S5

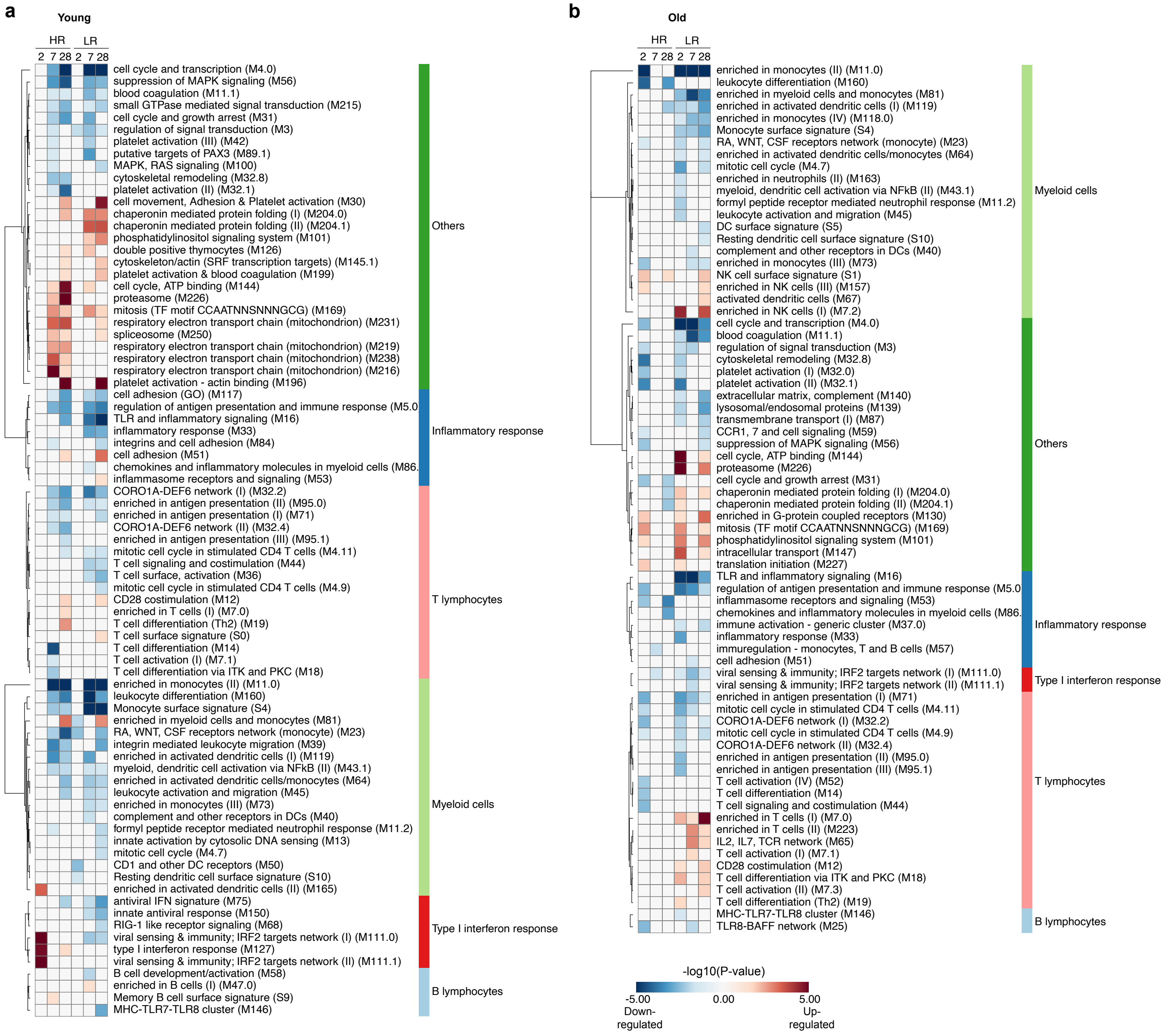
a
Young
HR
LR
2 7 28 2 7 28
cell cycle and transcription (M4.0)
suppression of MAPK signaling (M56)
blood coagulation (M11.1)
small GTPase mediated signal transduction (M215)
cell cycle and growth arrest (M31)
regulation of signal transduction (M3)
platelet activation (III) (M42)
putative targets of PAX3 (M89.1)
MAPK, RAS signaling (M100)
cytoskeletal remodeling (M32.8)
platelet activation (II) (M32.1)
cell movement, Adhesion & Platelet activation (M30)
chaperonin mediated protein folding (I) (M204.0)
chaperonin mediated protein folding (II) (M204.1)
phosphatidylinositol signaling system (M101)
double positive thymocytes (M126)
cytoskeleton/actin (SRF transcription targets) (M145.1)
platelet activation & blood coagulation (M199)
cell cycle, ATP binding (M144)
proteasome (M226)
mitosis (TF motif CCAATNNSNNNGCG) (M169)
respiratory electron transport chain (mitochondrion) (M231)
spliceosome (M250)
respiratory electron transport chain (mitochondrion) (M219)
respiratory electron transport chain (mitochondrion) (M238)
respiratory electron transport chain (mitochondrion) (M216)
platelet activation - actin binding (M196)
cell adhesion (GO) (M117)
regulation of antigen presentation and immune response (M5.0
TLR and inflammatory signaling (M16)
inflammatory response (M33)
integrins and cell adhesion (M84)
cell adhesion (M51)
chemokines and inflammatory molecules in myeloid cells (M86.
inflammasome receptors and signaling (M53)
CORO1A-DEF6 network (I) (M32.2)
enriched in antigen presentation (II) (M95.0)
enriched in antigen presentation (I) (M71)
CORO1A-DEF6 network (II) (M32.4)
enriched in antigen presentation (III) (M95.1)
mitotic cell cycle in stimulated CD4 T cells (M4.11)
T cell signaling and costimulation (M44)
T cell surface, activation (M36)
mitotic cell cycle in stimulated CD4 T cells (M4.9)
CD28 costimulation (M12)
enriched in T cells (I) (M7.0)
T cell differentiation (Th2) (M19)
T cell surface signature (S0)
T cell differentiation (M14)
T cell activation (I) (M7.1)
T cell differentiation via ITK and PKC (M18)
enriched in monocytes (II) (M11.0)
leukocyte differentiation (M160)
Monocyte surface signature (S4)
enriched in myeloid cells and monocytes (M81)
RA, WNT, CSF receptors network (monocyte) (M23)
integrin mediated leukocyte migration (M39)
enriched in activated dendritic cells (I) (M119)
myeloid, dendritic cell activation via NFkB (II) (M43.1)
enriched in activated dendritic cells/monocytes (M64)
leukocyte activation and migration (M45)
enriched in monocytes (III) (M73)
complement and other receptors in DCs (M40)
formyl peptide receptor mediated neutrophil response (M11.2)
innate activation by cytosolic DNA sensing (M13)
mitotic cell cycle (M4.7)
CD1 and other DC receptors (M50)
Resting dendritic cell surface signature (S10)
enriched in activated dendritic cells (II) (M165)
antiviral IFN signature (M75)
innate antiviral response (M150)
RIG-1 like receptor signaling (M68)
viral sensing & immunity; IRF2 targets network (I) (M111.0)
type I interferon response (M127)
viral sensing & immunity; IRF2 targets network (II) (M111.1)
B cell development/activation (M58)
enriched in B cells (I) (M47.0)
Memory B cell surface signature (S9)
MHC-TLR7-TLR8 cluster (M146)
Others
Inflammatory response
T lymphocytes
Myeloid cells
Type I interferon response
B lymphocytes
b
Old
HR
LR
2 7 28 2 7 28
enriched in monocytes (II) (M11.0)
leukocyte differentiation (M160)
enriched in myeloid cells and monocytes (M81)
enriched in activated dendritic cells (I) (M119)
enriched in monocytes (IV) (M118.0)
Monocyte surface signature (S4)
RA, WNT, CSF receptors network (monocyte) (M23)
enriched in activated dendritic cells/monocytes (M64)
mitotic cell cycle (M4.7)
enriched in neutrophils (II) (M163)
myeloid, dendritic cell activation via NFkB (II) (M43.1)
formyl peptide receptor mediated neutrophil response (M11.2)
leukocyte activation and migration (M45)
DC surface signature (S5)
Resting dendritic cell surface signature (S10)
complement and other receptors in DCs (M40)
enriched in monocytes (III) (M73)
NK cell surface signature (S1)
enriched in NK cells (III) (M157)
activated dendritic cells (M67)
enriched in NK cells (I) (M7.2)
cell cycle and transcription (M4.0)
blood coagulation (M11.1)
regulation of signal transduction (M3)
cytoskeletal remodeling (M32.8)
platelet activation (I) (M32.0)
platelet activation (II) (M32.1)
extracellular matrix, complement (M140)
lysosomal/endosomal proteins (M139)
transmembrane transport (I) (M87)
CCR1, 7 and cell signaling (M59)
suppression of MAPK signaling (M56)
cell cycle, ATP binding (M144)
proteasome (M226)
cell cycle and growth arrest (M31)
chaperonin mediated protein folding (I) (M204.0)
chaperonin mediated protein folding (II) (M204.1)
enriched in G-protein coupled receptors (M130)
mitosis (TF motif CCAATNNSNNNGCG) (M169)
phosphatidylinositol signaling system (M101)
intracellular transport (M147)
translation initiation (M227)
TLR and inflammatory signaling (M16)
regulation of antigen presentation and immune response (M5.0
inflammasome receptors and signaling (M53)
chemokines and inflammatory molecules in myeloid cells (M86.
immune activation - generic cluster (M37.0)
inflammatory response (M33)
immuregulation - monocytes, T and B cells (M57)
cell adhesion (M51)
viral sensing & immunity; IRF2 targets network (I) (M111.0)
viral sensing & immunity; IRF2 targets network (II) (M111.1)
enriched in antigen presentation (I) (M71)
mitotic cell cycle in stimulated CD4 T cells (M4.11)
CORO1A-DEF6 network (I) (M32.2)
mitotic cell cycle in stimulated CD4 T cells (M4.9)
CORO1A-DEF6 network (II) (M32.4)
enriched in antigen presentation (II) (M95.0)
enriched in antigen presentation (III) (M95.1)
T cell activation (IV) (M52)
T cell differentiation (M14)
T cell signaling and costimulation (M44)
enriched in T cells (I) (M7.0)
enriched in T cells (II) (M223)
IL2, IL7, TCR network (M65)
T cell activation (I) (M7.1)
CD28 costimulation (M12)
T cell differentiation via ITK and PKC (M18)
T cell activation (II) (M7.3)
T cell differentiation (Th2) (M19)
MHC-TLR7-TLR8 cluster (M146)
TLR8-BAFF network (M25)
Myeloid cells
Others
Inflammatory response
Type I interferon response
T lymphocytes
B lymphocytes
-log10(P-value)
-5.00
0.00
5.00
Down-
regulated
Up-
regulated

Figure S5. Blood transcription module (BTM) signatures in high and low responders.

BTM signatures in (**a**) young and (**b**) older cohorts.

Figure S6

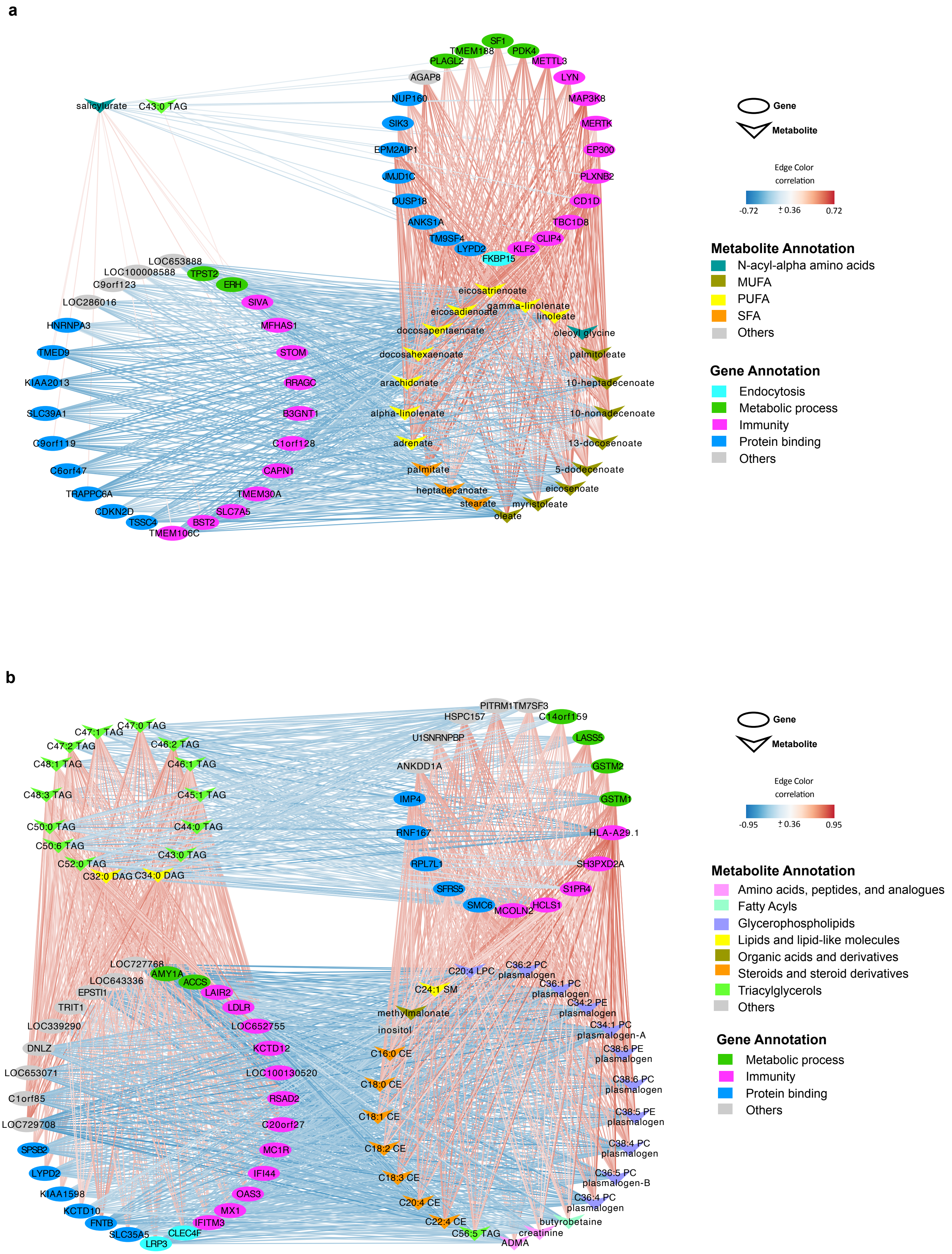
a
Gene
Metabolite
Edge Color
correlation
-0.72
± 0.36
0.72
Metabolite Annotation
N-acyl-alpha amino acids
MUFA
PUFA
SFA
Others
Gene Annotation
Endocytosis
Metabolic process
Immunity
Protein binding
Others
salicylurate
C43:0 TAG
SF1
TMEM188
PDK4
PLAGL2
METTL3
AGAP8
LYN
NUP160
MAP3K8
SIK3
MERTK
EPM2AIP1
EP300
JMJD1C
PLXNB2
DUSP18
CD1D
ANKS1A
TBC1D8
TM9SF4
CLIP4
LYPD2
KLF2
FKBP15
LOC653888
LOC100008588
TPST2
C9orf123
ERH
LOC286016
SIVA
HNRNPA3
MFHAS1
TMED9
STOM
KIAA2013
RRAGC
SLC39A1
B3GNT1
C9orf119
C1orf128
C6orf47
CAPN1
TRAPPC6A
TMEM30A
CDKN2D
SLC7A5
TSSC4
BST2
TMEM106C
eicosatrienoate
eicosadienoate
gamma-linolenate
linoleate
docosapentaenoate
oleoyl glycine
docosahexaenoate
palmitoleate
arachidonate
10-heptadecenoate
alpha-linolenate
10-nonadecenoate
adrenate
13-docosenoate
palmitate
5-dodecenoate
heptadecanoate
eicosenoate
stearate
myristoleate
oleate
b
Gene
Metabolite
Edge Color
correlation
-0.95
± 0.36
0.95
Metabolite Annotation
Amino acids, peptides, and analogues
Fatty Acyls
Glycerophospholipids
Lipids and lipid-like molecules
Organic acids and derivatives
Steroids and steroid derivatives
Triacylglycerols
Others
Gene Annotation
Metabolic process
Immunity
Protein binding
Others
C47:0 TAG
C47:1 TAG
C47:2 TAG
C46:2 TAG
C48:1 TAG
C46:1 TAG
C48:3 TAG
C45:1 TAG
C50:0 TAG
C44:0 TAG
C50:6 TAG
C43:0 TAG
C52:0 TAG
C32:0 DAG
C34:0 DAG
PITRM1
TM7SF3
HSPC157
C14orf159
U1SNRNPBP
LASS5
ANKDD1A
GSTM2
IMP4
GSTM1
RNF167
HLA-A29.1
RPL7L1
SH3PXD2A
SFRS5
S1PR4
SMC6
MCOLN2
HCLS1
LOC727768
AMY1A
ACCS
LOC643336
EPSTI1
LAIR2
TRIT1
LDLR
LOC339290
LOC652755
DNLZ
KCTD12
LOC653071
LOC100130520
C1orf85
RSAD2
LOC729708
C20orf27
SPSB2
MC1R
LYPD2
IFI44
KIAA1598
OAS3
KCTD10
MX1
FNTB
IFITM3
SLC35A5
CLEC4F
LRP3
C20:4 LPC
C36:2 PC plasmalogen
C24:1 SM
C36:1 PC plasmalogen
C34:2 PE plasmalogen
methylmalonate
inositol
C34:1 PC plasmalogen-A
C16:0 CE
C38:6 PE plasmalogen
C18:0 CE
C38:6 PC plasmalogen
C18:1 CE
C38:5 PE plasmalogen
C18:2 CE
C38:4 PC plasmalogen
C18:3 CE
C36:5 PC plasmalogen-B
C20:4 CE
C36:4 PC plasmalogen
C22:4 CE
butyrobetaine
C56:5 TAG
creatinine
ADMA

Figure S6. Integrated metabolomic and transcriptomic correlation and network in young and older high responders. Comprehensive correlation networks of genes and metabolites in young (**a**) and older (**b**) high responders (correlation cut-off: 0.40).
